# Interactive impact of childhood maltreatment, depression, and age on cortical brain structure: mega-analytic findings from a large multi-site cohort

**DOI:** 10.1017/S003329171900093X

**Published:** 2019-05-14

**Authors:** Leonardo Tozzi, Lisa Garczarek, Deborah Janowitz, Dan J. Stein, Katharina Wittfeld, Henrik Dobrowolny, Jim Lagopoulos, Sean N. Hatton, Ian B. Hickie, Angela Carballedo, Samantha J. Brooks, Daniella Vuletic, Anne Uhlmann, Ilya M. Veer, Henrik Walter, Robin Bülow, Henry Völzke, Johanna Klinger-König, Knut Schnell, Dieter Schoepf, Dominik Grotegerd, Nils Opel, Udo Dannlowski, Harald Kugel, Elisabeth Schramm, Carsten Konrad, Tilo Kircher, Dilara Jüksel, Igor Nenadić, Axel Krug, Tim Hahn, Olaf Steinsträter, Ronny Redlich, Dario Zaremba, Bartosz Zurowski, Cynthia H.Y. Fu, Danai Dima, James Cole, Hans J. Grabe, Colm G. Connolly, Tony T. Yang, Tiffany C. Ho, Kaja Z. LeWinn, Meng Li, Nynke A. Groenewold, Lauren E. Salminen, Martin Walter, Alan N Simmons, Theo G.M. van Erp, Neda Jahanshad, Bernhard T. Baune, Nic J.A. van der Wee, Marie-Jose van Tol, Brenda W.J.H. Penninx, Derrek P. Hibar, Paul M. Thompson, Dick J. Veltman, Lianne Schmaal, Thomas Frodl

**Affiliations:** 1 Department of Psychiatry and Psychotherapy, Otto von Guericke University, Magdeburg, Germany; 2 Department of Psychiatry and Behavioral Sciences, Stanford University, California, USA; 3 Department of Psychiatry and Psychotherapy, University Medicine Greifswald, Greifswald, Germany; 4 SAMRC Unit on Risk & Resilience in Mental Disorders, UCT Department of Psychiatry and Mental Health, Cape Town, South Africa; 5 German Center for Neurodegenerative Diseases (DZNE), Site Rostock/Greifswald, Germany; 6 Brain and Mind Centre, University of Sydney, Camperdown, Australia; 7 Sunshine Coast Mind and Neuroscience – Thompson Institute, Queensland, Australia; 8 Trinity College Institute of Neuroscience, Trinity College Dublin, Ireland; 9 School of Natural Sciences and Psychology, Liverpool John Moores University, Liverpool, UK; 10 Department of Psychiatry, University of Vermont, Burlington, VT, USA; 11 Division of Mind and Brain Research, Department of Psychiatry and Psychotherapy CCM, Charité – Universitätsmedizin Berlin, corporate member of Freie Universität Berlin, Humboldt-Universität zu Berlin, and Berlin Institute of Health, Berlin, Germany; 12 Institute for Diagnostic Radiology and Neuroradiology, University Medicine Greifswald, Germany; 13 Institute for Community Medicine, University Medicine Greifswald, and Center of Cardiovascular Research (DZHK), Germany, partner site Greifswald; 14 Department of General Psychiatry, University Hospital Heidelberg, Germany; 15 Department of Psychiatry and Psychotherapy, University Medical Center Göttingen, Göttingen, Germany; 16 Department of Psychiatry and Psychotherapy, Asklepios Fachklinikum Göttingen, Göttingen, Germany; 17 Department of Psychiatry and Psychotherapy, University of Bonn, Germany, and Department of Psychiatry and Psychotherapy, Vitos Weil-Lahn, Hesse, Germany; 18 Department of Psychiatry and Psychotherapy, University of Münster, Germany; 19 Institute of Clinical Radiology, University of Münster, Germany; 20 Department of Psychiatry and Psychotherapy, Medical Center, University of Freiburg, Germany; 21 Psychiatric University Clinic, Basel, Switzerland; 22 Department of Psychiatry and Psychotherapy, Agaplesion Diakoniklinikum, Rotenburg, Germany; 23 Department of Psychiatry and Psychotherapy, Philipps-University Marburg, Germany; 24 Core Facility Brain Imaging, Faculty of Medicine, Philipps-University of Marburg, Germany; 25 Center for Integrative Psychiatry, University of Lübeck, Lübeck, Germany; 26 School of Psychology, College of Applied Health and Communities, University of East London, London, UK; 27 Centre for Affective Disorders, Institute of Psychiatry, Psychology and Neuroscience, King’s College London, London, UK; 28 Department of Psychology, School of Arts and Social Sciences, City, University of London, London, UK; 29 Department of Neuroimaging, Institute of Psychiatry, Psychology and Neuroscience, King’s College London, London, UK; 30 Department of Psychiatry & Langley Porter Psychiatric Institute, UCSF Weill Institute for Neurosciences, University of California, San Francisco, USA; 31 Department of Biomedical Sciences, Florida State University Tallahassee, FL, USA; 32 Department of Psychiatry, Division of Child and Adolescent Psychiatry, University of California, San Francisco (UCSF), USA; 33 Department of Psychology and Department of Psychiatry & Behavioral Sciences, Stanford University, Stanford, CA, USA; 34 Leibniz Institute for Neurobiology, Magdeburg, Germany; 35 Imaging Genetics Center, Mark and Mary Stevens Neuroimaging and Informatics Institute, Keck School of Medicine of University of California, Marina del Rey, CA, USA; 36 Department of Psychiatry and Psychotherapy, University of Tuebingen, Germany; 37 VA San Diego Healthcare, San Francisco, CA, USA; 38 School of Medicine, Department of Psychiatry and Human Behavior, University of California, Irvine, CA, USA; 39 Clinical Translational Neuroscience Laboratory, Department of Psychiatry and Human Behavior, University of California, Irvine, CA, USA; 40 Discipline of Psychiatry, School of Medicine, University of Adelaide, SA 5005 Adelaide, Australia; 41 Department of Psychiatry, Melbourne Medical School, The University of Melbourne, VIC 3010 Melbourne, Australia; 42 The Florey Institute of Neuroscience and Mental Health, The University of Melbourne, Melbourne, Australia; 43 Department of Psychiatry, Leiden Institute for Brain and Cognition, Leiden University Medical Center, Leiden, The Netherlands; 44 Department of Biomedical Sciences of Cells and Systems, Cognitive Neuroscience Center, University Medical Center Groningen, University of Groningen, Groningen, The Netherlands; 45 Department of Psychiatry and Neuroscience Campus Amsterdam, VU University Medical Center, Amsterdam, The Netherlands; 46 Orygen, The National Centre of Excellence in Youth Mental Health, Melbourne, Australia; 47 Centre for Youth Mental Health, The University of Melbourne, Melbourne, Australia; 48 German Center of Neurodegenerative Diseases (DZNE), Site Magdeburg, Germany

**Keywords:** Childhood maltreatment, cortical thickness, ENIGMA, major depressive disorder

## Abstract

**Background.:**

Childhood maltreatment (CM) plays an important role in the development of major depressive disorder (MDD). The aim of this study was to examine whether CM severity and type are associated with MDD-related brain alterations, and how they interact with sex and age.

**Methods.:**

Within the ENIGMA-MDD network, severity and subtypes of CM using the Childhood Trauma Questionnaire were assessed and structural magnetic resonance imaging data from patients with MDD and healthy controls were analyzed in a mega-analysis comprising a total of 3872 participants aged between 13 and 89 years. Cortical thickness and surface area were extracted at each site using *FreeSurfer*.

**Results.:**

CM severity was associated with reduced cortical thickness in the banks of the superior temporal sulcus and supramarginal gyrus as well as with reduced surface area of the middle temporal lobe. Participants reporting both childhood neglect and abuse had a lower cortical thickness in the inferior parietal lobe, middle temporal lobe, and precuneus compared to participants not exposed to CM. In males only, regardless of diagnosis, CM severity was associated with higher cortical thickness of the rostral anterior cingulate cortex. Finally, a significant interaction between CM and age in predicting thickness was seen across several prefrontal, temporal, and temporo-parietal regions.

**Conclusions.:**

Severity and type of CM may impact cortical thickness and surface area. Importantly, CM may influence age-dependent brain maturation, particularly in regions related to the default mode network, perception, and theory of mind.

## Introduction

According to the Centers for Disease Control and Prevention, childhood maltreatment (CM) is defined as ‘any act or series of acts of commission or omission by a parent or other caregiver that results in harm, potential for harm, or threat of harm to a child’ (Leeb et al., 2008). CM may be physical, sexual or emotional and may result in inadequate environmental input (e.g. deprivation or neglect) or excessive harmful input (Sheridan and McLaughlin, 2014). About one-quarter of all adults have encountered CM in their life (Fu et al., 2018) and this statistic may even be higher as a history of childhood adversity is likely under-reported. In fact, a recent meta-analysis has found that over half of children globally had experienced violence in just the past year alone (Hillis et al., 2016). Given the prevalence of CM, this is especially alarming as CM is strongly associated with a wide range of adverse consequences, not only causing suffering in the immediate aftermath, but also long-term detrimental effects to mental and physical health. For example, children with a history of CM are more prone to smoking and obesity, as well as of being perpetrators and victims of violence (WHO, 2016, November). Also, both prospective and retrospective reports of maltreatment were found to be associated with adult psychiatric disorders in a recent study, though the strongest associations were found when maltreatment was retrospectively self-reported (Newbury et al., 2018). In that study, it was also shown that young adults who recall being maltreated have a particularly elevated risk for psychopathology. Notably, CM is one of the strongest factors in the development of major depressive disorder (MDD) (Bernet and Stein, 1999), the leading cause of disability worldwide according to the World Health Organization, with increasing rates over the past decade (WHO, 2016, November).

As both CM and MDD have a high incidence in the general population, the interplay between these two phenomena is important to investigate, both for prevention and treatment. Depressed patients with CM, for example, respond more poorly to antidepressant treatment than those without CM (Nanni et al., 2012). CM and MDD may be causally linked, as MDD is a disorder characterized by pathological responses to stress (Frodl et al., 2008). In experimental studies, chronic social stress induces glucocorticoid-mediated pyramidal dendrite retraction in the hippocampus and changes in dendrite arborization in the prefrontal cortex (PFC) (Woolley et al., 1990; Magarinos et al., 1996; Wellman, 2001; Kole et al., 2004), which may be associated with the behavioral manifestations of stress-related disorders like MDD (Macqueen and Frodl, 2010). Therefore, based on the extant literature, one hypothesis is that CM in humans acts as a chronic stressor contributing to changes of brain structure and function, which in turn may increase vulnerability to psychiatric disorders such as MDD. Supporting this theory, CM was found to be associated with reduced brain volumes in the amygdala, PFC, and cerebellum (Frodl et al., 2010; Edmiston et al., 2011; Dannlowski et al., 2012; Teicher et al., 2016) – regions also reported to be affected in MDD (Wise et al., 2016; Schmaal et al., 2017). A correlation between CM and medial prefrontal gray matter volume was also detected irrespective of diagnosis with MDD or anxiety (van Harmelen et al., 2010). Finally, in an ENIGMA-MDD mega-analysis focusing on subcortical structures, CM was found to be associated with lower caudate volume in females. Those alterations were more strongly associated with emotional and physical neglect than with other forms of CM (Frodl et al., 2017).

Research in animals and humans also suggests important distinctions between types of CM on brain structure. Specifically, researchers theorized that experiences characterized by deprivation (e.g. emotional and physical neglect) compared with experiences characterized by threat (e.g. emotional abuse and physical violence) lead to different effects on neuronal development (McLaughlin et al., 2014a). A community study in 287 adolescents showed that exposure to threat and violence was associated with automatic emotion regulation deficits, but not cognitive control disturbances. In contrast, exposure to poverty was associated with worse cognitive control, but no deficits automatic emotion regulation. On the other hand, both violence and poverty predicted poor inhibition in an emotional context (Lambert et al., 2017). Interestingly, children exposed to severe deprivation in the form of institutional rearing exhibited widespread cortical thinning in the superior and inferior parietal cortex (McLaughlin et al., 2014b), and children exposed to neglect often have deficits in language abilities (Farah et al., 2006). Individuals with a history of deprivation showed smaller gray matter volumes compared with individuals with a history of abuse in the fusiform gyrus and the middle occipital gyrus (Everaerd et al., 2016). Therefore, exploring effects from different types of CM on brain structure was an important goal of the current study.

In this mega-analysis, we first aimed to investigate the association between CM severity and cortical brain structure in MDD patients and healthy subjects. We hypothesized that more severe CM would be related to lower cortical thickness and surface area, especially of the OFC, ACC, medial PFC, and insula – regions affected in adult MDD (Fischl et al., 2002) and involved in emotion regulation (Desikan et al., 2006). We also hypothesized that MDD patients with a more severe history of CM would show smaller cortical brain measures than healthy controls (HC) with a similar history of CM. Prior studies detected effects of CM on dorsomedial PFC volume irrespective of diagnosis, but did not fully consider the severity of CM. Besides severity, we also investigated the relationship between different types of CM and brain structure. Furthermore, we hypothesized changes to be more prominent in females than males (Frodl et al., 2017) and thus investigated the interactions between sex and CM on brain structure. Finally, given the large sample size and wide age range, we aimed to explore the interactive effects of CM with age on brain structure.

## Methods and materials

### Samples

In the current study, 12 international sites participating in the ENIGMA MDD Workgroup with information on CM agreed to participate in the Childhood Adversity Subgroup. Detailed demographics and clinical characteristics for each sample may be found in eTables 1 and 2. Most studies used SCID-1, CIDI, or another form of a standardized interview (eTable 3). Exclusion criteria for study enrollment are given in eTable 3. In total, we analyzed data from 3872 participants: 1284 patients with a lifetime history of MDD and 2588 HC. All participating sites obtained approval from local institutional review boards and ethics committees. In addition, this mega-analysis was approved by the ethics board of the medical faculty of the Otto von Guericke University Magdeburg, Germany. All study participants provided written consent at their local site. In the case of adolescent participants, parent/legal guardian provided written consent and the adolescent provided written assent.

### Assessment

Severity of CM (CM-severity) was measured across all sites with the Childhood Trauma Questionnaire (CTQ) (Bernstein et al., 1994). The short form of the CTQ is a standardized self-report instrument consisting of 28 items containing five major subscales of CM. Each one also features a cut-off to determine the presence of emotional (⩾12), physical (⩾10), and sexual abuse (⩾8) or emotional (⩾15) and physical neglect (⩾10). Three additional items provide information on responders’ tendencies toward minimization and denial. For our analyses, we assessed CM in two ways. First, based on a score above the cut-off for at least one of the abuse or neglect subscales, we divided our participants into four groups (CM-type): no CM, neglect (no abuse), abuse (no neglect), abuse + neglect. In a second analysis, we explored the effect of CTQ total sum score as a continuous variable (CM-severity).

Severity of depressive symptoms at the time of scanning was measured in some sites with the Hamilton Depression Questionnaire (HDRS-17), in others, the Beck Depression Inventory (BDI-II) or Inventory of Depressive Symptomatology-Self Report (IDS-SR) was used. Age of onset and antidepressant medication use at the time of scan were also recorded in 11 and 12 sites, respectively.

### Image processing and analysis

Participants all underwent structural T1-weighted MRI brain scans locally at each site, where scans were analyzed using the fully-automated and validated segmentation software FreeSurfer (version 5.0 or higher) (Fischl et al., 2002). Image acquisition parameters and software descriptions for each sample are given in eTable 4. Deep brain structure volumes were extracted and visually inspected for segmentation accuracy. Parcellations for cortical thickness and surface area of 68 (34 left and 34 right) regions based on the Desikan–Killiany atlas (Desikan et al., 2006) and left and right hemisphere measures were derived and visually inspected for accuracy following a protocol designed to facilitate harmonized image analysis across multiple sites (http://enigma.ini.usc.edu/protocols/imaging-protocols/). Association between CM and subcortical measures were previously published (Frodl et al., 2017).

### Statistical framework of mega-analysis

Statistical analyses were performed using SPSS statistics (version 24).

We performed ANOVAs, or Kruskal–Wallis tests as appropriate, to compare at scan, age at MDD onset, clinical severity of depression, and CTQ scores between groups and cohorts. The χ^2^ tests were used to analyze differences between frequencies of males and females.

Then, we built generalized estimating equation (GEE) models with thickness or surface area of each region as the dependent variable. Our models had a linear scale response. All participants were included, irrespective of diagnosis. The independent between-subject variable CM was defined in two ways: as the factor CM-type (0 = no CM, 1 = neglect, 2 = abuse, 3 = neglect + abuse) or as CM-severity (continuous: total CTQ). Each of these variables was included in separate models. In all models, we included the between-subjects factors diagnosis (factor: 1 = patients, 0 = HC), sex (factor: 0 = males, 1 = females), and the within-subject factor hemisphere (left, right). Age (continuous), neuroimaging cohort (factor), and total intracranial volume (continuous) were used as between-subject covariates. *FreeSurfer* version and scanner type were comprised in the factor neuroimaging cohort. As we did not expect CM-severity effects to be lateralized, hemisphere was only included as a main effect. Our prior research showed differential effects of CM-severity in predicting the volume of subcortical structures depending on sex and MDD diagnosis (Frodl et al., 2017). Therefore, we explored in our models all possible interactions between CM, sex, and diagnosis for both surface and thickness. To assess the effect of CM across all brain areas, we first ran analyses on the total thickness and surface across all regions respectively, adding region as a within-subject factor. Then, we repeated the process for each region individually. Finally, we explored the interaction between age and severity of CM while keeping all other terms in the model as the main effects.

In all models, Wald χ^2^ tests were used to assess the significance of each term. To account for multiple tests (34 regions), a false discovery rate (FDR) correction was computed on the resulting *p*-values. Findings were considered significant if *p*_FDR_<0.05. Any significant interactive effects resulting from the models described above were followed up with post-hoc testing.

#### Investigation of clinical confounds

A subset of our MDD cohort (*N* = 966) had more detailed clinical information and allowed us to explore additional potential confounding effects. Therefore, we investigated if the thickness or surface of all regions was significantly predicted by clinical severity (continuous: BDI total score, since HAM-D was available only for a minor subset of participants), recurrence (factor: 0 = first episode, 1 = recurrent episode), current antidepressant use (factor: 0 = no, 1 = yes), age of depression onset (continuous). Information about clinical remission was not provided by all sites; therefore, for this analysis, we defined it as current BDI⩽12 (Riedel et al., 2010). We then built GEE models that featured these measures as predictors (main effects), together with CM (severity or type), age, sex, site, hemisphere, and total intracranial volume. Detailed information on this subsample is presented in eTable 14.

## Results

### Demographics

For details on participant’s demographics and clinical features, see Table 1. Overall, data significantly differed between centers with respect to sex, age, CTQ scores, and clinical features (see eTable 5). Frequency of co-occurrence between abuse and neglect is 12.7%. CM severity is influenced by abuse and neglect to a similar extent (*β* = 0.41 and *β* = 0.42, respectively).

### Cortical thickness

#### Main effects of CM-severity

A summary of all significant findings is reported in Table 2. For an overview of the results of the models run on each region, see eTable 6.

**Fig. 1.**
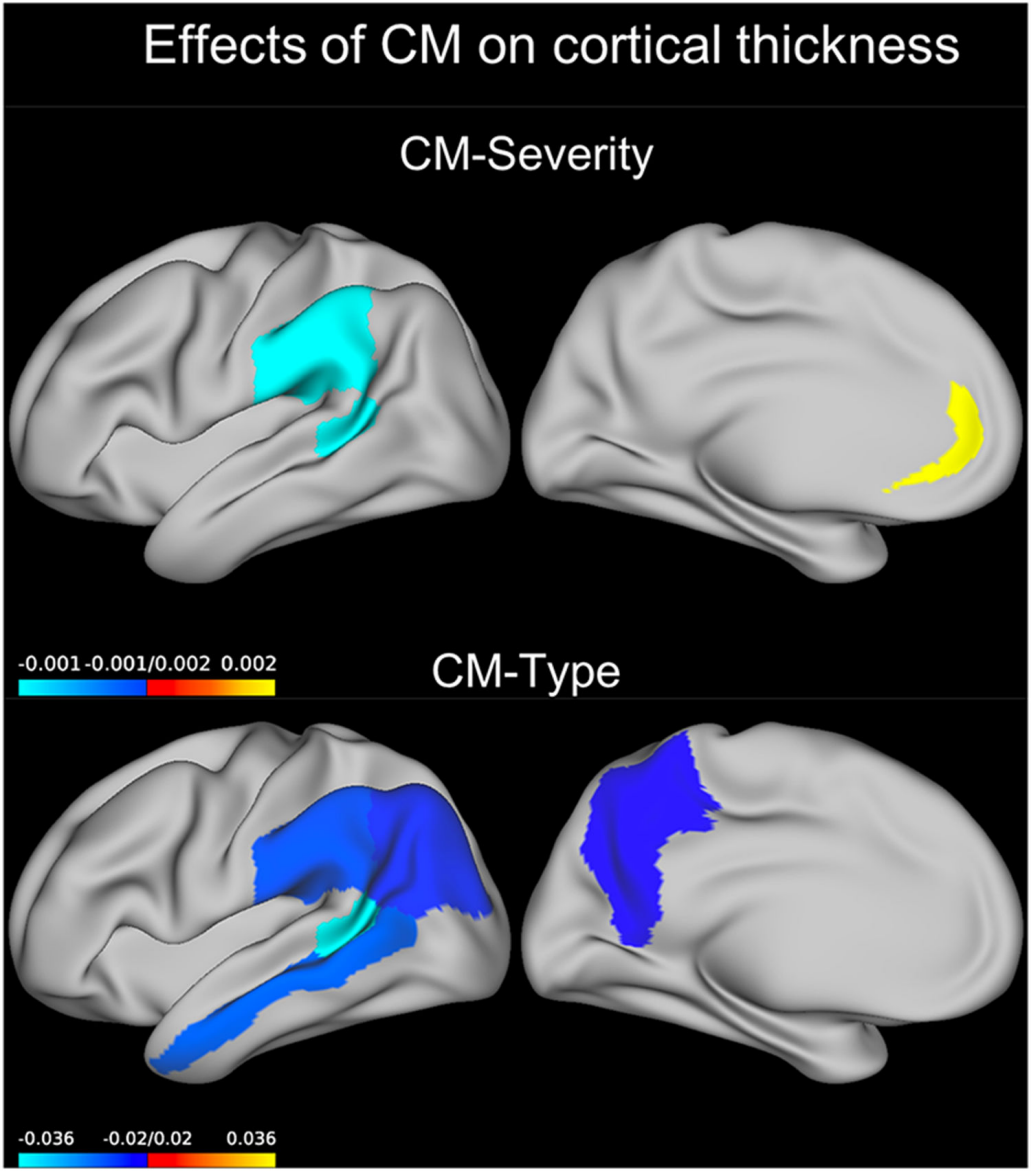
Effect of CM predicting cortical thickness. Coefficients for the GEE model term CM severity or those for the neglect + abuse group compared to the no-CM group are plotted on an inflated left brain hemisphere (effects were bilateral). Only the neglect + abuse group was different from the no-CM group. CM, childhood maltreatment; GEE, generalized estimating equations.

We detected a significant main effect showing an inverse relation between CM-severity and thickness of the banks of the superior temporal sulcus (Wald χ^2^ = 14.583, *p*_FDR_ = 0.033, *B* = −0.001, Fig. 1). A significant main effect of CM was also present on the thickness of the supramarginal gyrus (Wald χ^2^ = 8.889, *p*_FDR_ = 0.049, *B* = −0.001, Fig. 1).

#### CM-severity and sex interaction

When considering all regions, the interaction between CM-severity and sex was significant (Wald χ^2^ = 5.220, *p* = 0.022). Dividing the data by sex, post-hoc analyses showed a significant negative effect of CM-severity on cortical thickness in females (Wald χ^2^ = 4.861, *p* = 0.027, *B* = −0.000649), but not in males (Wald-χ^2^ = 1.287, *p* = 0.257, *B* = −0.000136).

When running models for each region separately, we found a significant interaction between CM-severity and sex on the cortical thickness of the rostral anterior cingulate cortex (Wald χ^2^ = 13.556, *p*_FDR_ = 0.008). Post-hoc analysis revealed a significant positive effect of CM-severity on the cortical thickness of this region in males (Wald χ^2^ = 14.426, *p* < 0.001, *B* = 0.002, Fig. 1) but not in females (Wald χ^2^ = 3.174, *p* = 0.075, *B* = −0.0006).

#### CM-severity and age interaction

When considering all regions, a significant interaction between age and severity of CM was detected (Wald χ^2^ = 11.105, *p* = 0.001, *B* = −0.000035).

**Fig. 2.**
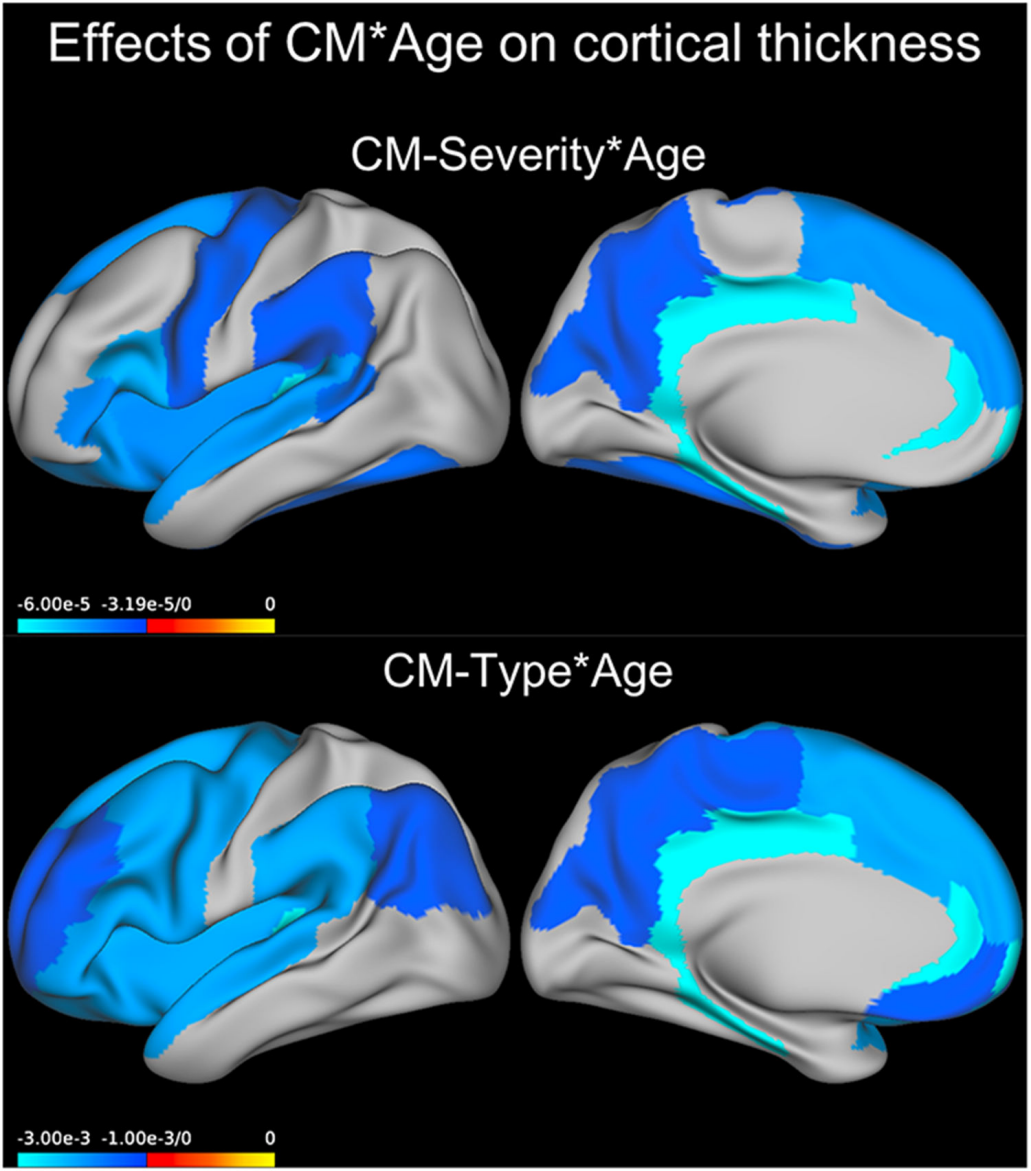
Effect of CM × Age predicting cortical thickness. Coefficients for the GEE model term CM severity × Age or those for Age in the abuse + neglect group compared to the no-CM group are plotted on an inflated left brain hemisphere (effects were bilateral). CM, childhood maltreatment, GEE, generalized estimating equations.

Models ran for each region separately indicated that this interaction between age and severity of CM was significant across all participants in the rostral anterior cingulate, isthmus of the cingulate, posterior cingulate, lateral orbitofrontal gyrus, parahippocampal gyrus, inferior frontal gyrus (IFG) pars opercularis, IFG pars triangularis, superior frontal gyrus, banks of the superior temporal sulcus, cuneus, fusiform gyrus, insula, precentral gyrus, precuneus, supramarginal gyrus, and transverse temporal gyrus (see Table 3, eTable 7, Fig. 2).

#### Main effects of CM-type

We found a significant main effect of CM type (eTable 10) in the banks of the superior temporal sulcus (Wald χ^2^ = 19.888, *p*_FDR_ = 0.006), inferior parietal lobe (Wald χ^2^ = 15.273, *p*_FDR_ = 0.023), middle temporal lobe (Wald χ^2^ = 12.123, *p*_FDR_ = 0.048), precuneus (Wald χ^2^ = 15.325, *p*_FDR_ = 0.023), and supramarginal gyrus (Wald χ^2^ = 13.990, *p*_FDR_ = 0.026). In all cases, the neglect + abuse group had lower mean thickness values compared to the no CM group (all *p* < 0.01, Fig. 1) and there was no difference between the abuse only as well as neglect only CM types and the no CM group.

#### CM-type and age interaction

The interaction between age and type of CM was significant across all participants for most regions (see Table 3, eTable 11). In all cases, the effects of age were more negative in the neglect + abuse group compared to the CM group (all *p* < 0.05).

### Cortical surface area

A summary of all significant findings is reported in Table 2. For an overview of the results of the models run on each region, see eTable 8.

#### Main effects of CM-severity

**Fig. 3.**
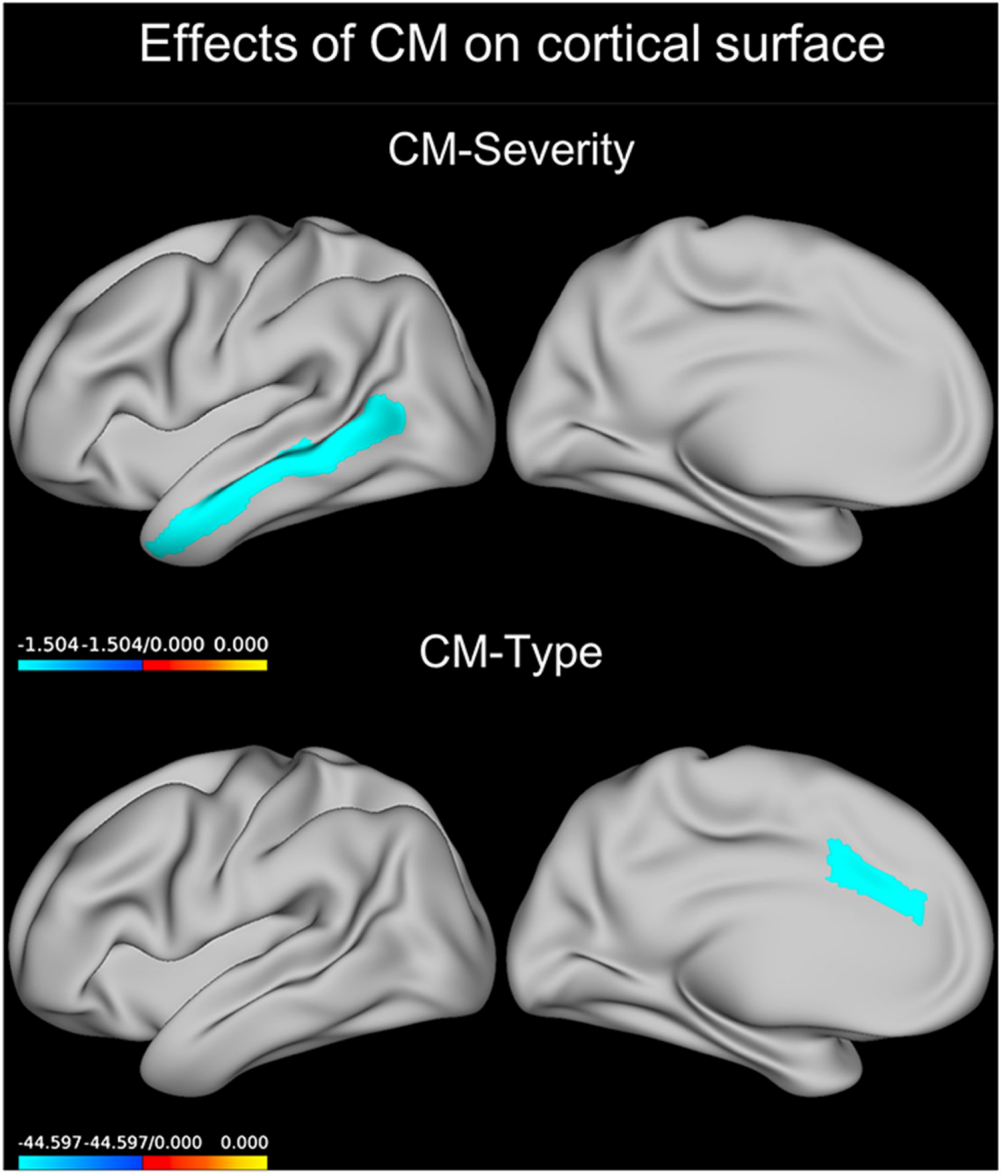
Effect of CM predicting cortical surface. Coefficients for the GEE model term CM severity or those for the abuse only group compared to the no-CM group are plotted on an inflated left brain hemisphere (effects were bilateral). The neglect group showed a similar result in the same region. CM, childhood maltreatment; GEE, generalized estimating equations.

Across all regions, a negative main effect of CM-severity on the cortical surface area was observed (Wald χ^2^ = 4.413, *p* = 0.036, *B* = −0.414). When running separate models for each region (eTable 8), we detected a significant inverse main effect of CM-severity on the surface area of the middle temporal gyrus (Wald χ^2^ = 12.368, *p*_FDR_ = 0.015, *B* = −1.504, Fig. 3).

#### CM-type, diagnosis, and sex interaction

We found a significant interaction between CM type, diagnosis, and sex (eTable 12) in the caudal anterior cingulate (Wald χ^2^ = 17.807, *p*_FDR_ < 0.001). Post-hoc testing revealed that, in depressed males, those having suffered from either abuse or neglect had a lower average cortical surface area of the caudal anterior cingulate cortex than those who had no history of CM (*p* = 0.003 and *p* = 0.017, respectively, Fig. 3).

#### CM-severity/type and age interaction

We found no effects of the interaction between CM-severity or CM type and age in predicting cortical surface area (eTable 9).

#### Investigation of clinical confounds

Our post-hoc investigation in a subset of patients with detailed information showed no significant effects of clinical variables on thickness or surface (all *p* > 0.05). See eTables 15-18 for the model effects.

## Discussion

This study represents the largest effort worldwide to investigate the association between CM and cortical brain structure in a large sample of MDD patients and healthy subjects. We found that CM has a subtle but widespread association with cortical thickness and surface area, which is likely influenced by sex and age.

Two procedures of describing CM were used. First, the dimensional measure of CM severity allowed for continuous analysis. Second, the categorical classification of CM into no CM, only neglect, only abuse, and both abuse and neglect, allowed for an analysis of the type of CM. It should be highlighted that participants exposed to both neglect and abuse also had higher total CM values.

Severity of CM was associated with lower mean cortical surface area regardless of the region across all MDD patients and HC. In women, higher severity of CM was also associated with thinner thickness across all regions. These findings are consistent with prior research showing widespread effects of severity of CM on the brain irrespective of psychopathological status (Chaney et al., 2014).

Regardless of diagnosis, when individual regions were investigated, this effect of CM severity survived correction for multiple testing in temporal and temporo-parietal regions. Specifically, participants with higher CM severity had significantly thinner cortex in the banks of the superior temporal sulcus and the supramarginal gyrus. When considering the type of abuse, high severity of CM, represented by concurrent childhood neglect and abuse, was once again associated with reduced thickness in these two areas and additionally in the precuneus, middle temporal lobe, and inferior parietal cortex. Moreover, participants with higher severity of CM also showed a smaller surface area of the middle temporal gyrus. The magnitude of these negative effects on thickness and surface area pointed toward a reduction around 0.001 mm of thickness and around 0.4–1.5 mm^2^ of surface with each one-point increase in CTQ score depending on the brain region. For example, an increase of 100 points in the CTQ scale would be associated with a 0.1 mm (or 4%) decrease of cortical thickness in the banks of the superior temporal sulcus. The middle temporal lobe is thought to be essential for our ability to understand actions and semantic associations (Davey et al., 2016). One possibility is that CM may lead to difficulties in semantic retrieval through alterations in regions of temporal cortex and the default mode network. Indeed, other studies have also demonstrated that individuals with higher severity of CM showed reduced cortical surface on the left middle temporal area and lingual gyrus (Kelly et al., 2013). In contrast, in a study of adolescents and young adults exposed to CM, increased cortical volume was observed in the left inferior and middle temporal gyri relative to HC (Lim et al., 2018). In the present study, the other regions we report showing an impact from CM type are located in the temporo-parietal area and around the temporo-parietal junction; both of these regions play a role in the theory of mind processing which is important during daily social interactions (Saxe and Kanwisher, 2003). Deficits in these areas might suggest a disadvantage for subjects with a history of CM, in particular those with increased severity and more types of CM. No significant main effects of severity of CM were detected for other regions we hypothesized to be vulnerable. For example, prior studies found a significant main effect of CM in different regions such as fronto-limbic areas, visual cortex, and cerebellum (Kelly et al., 2013; Yang et al., 2017). This might be due to smaller sample sizes and more homogeneity in prior studies: in our analysis, which features a larger sample size of *N* = 3872, we detected an overall effect of severity of CM on the whole cortex with some prominence in the temporal and temporo-parietal regions.

Another interesting finding was that males, but not females, with a more severe history of CM, regardless of diagnosis, showed distinctly thicker rostral anterior cingulate cortices. These results suggest sex differences in the effects of CM on the structure of this region (Fallucca et al., 2011; Canu et al., 2015). The anterior cingulate cortex is involved in emotional and inhibitory processes (Garavan et al., 2006; Steele et al., 2013). Thus, males seem to be particularly sensitive to CM with regards to the thickness in a region relevant to emotion regulation and might show a reactive increase of thickness. Whether this thickness increase of the rostral ACC is adaptive cannot be effectively addressed in the current sample, as longitudinal data and resiliency measures were not available for the bulk of the cohort. In this context, it is interesting that male patients with a history of neglect and abuse had significantly smaller surface areas of the caudal ACC compared to those without CM, pointing toward a negative effect of CM in the caudal ACC in participants who developed MDD. In healthy subjects, such an effect of CM was not seen in the caudal ACC and we could tentatively ascribe to resilience (Feder et al., 2009).

### Childhood maltreatment by age interaction

A novel finding detected in our secondary analysis was that CM severity and age interacted regardless of diagnosis to predict the thickness of several cortical regions involved in emotional processing, such as portions of the cingulate, orbitofrontal, insular, dorsolateral prefrontal, and medial prefrontal cortices. In these areas, older people including patients and controls with higher CM severity had lower cortical thickness. The orbitofrontal and dorsomedial prefrontal cortices can be seen as separate networks interacting closely with limbic structures, but also showing cortico-cortical interconnections with each other (Ongur and Price, 2000; Phillips et al., 2008). These regions allow the brain to process emotionally salient information and help with the regulation of emotional behavior (Phillips et al., 2008). The insula is closely interconnected with the orbitofrontal cortex and is involved in emotion and executive processing as well as working memory (Levens and Phelps, 2010). The cingulate cortex is also well known to have cognitive and emotional functions: its dorsal parts are involved in emotion evaluation, whereas the ventral parts and the dorsomedial PFC are involved in emotion regulation (Etkin et al., 2011). Overall, these results are consistent with previous studies showing that CM-severity impacts regions involved in emotion regulation, including the insula (Teicher et al., 2014).

Also, our cross-sectional data suggest that cortical thickness might decrease more rapidly with age in individuals with a more severe history of CM, although this still needs to be confirmed by longitudinal analyses. It will be critical for future studies to assess the effects of abuse across multiple time points and to consider such abuse in relation to the age of participants.

### Diagnosis and CM interactions

In the present study, no effect of diagnosis was detected and we also did not find a significant interaction between diagnosis and CM. It is possible that we could not replicate the main effect of MDD diagnosis on OFC and ACC thickness because of the smaller sample size of the current study (overall *N* = 3872) compared to the sample size in our previous ENIGMA MDD meta-analysis that focused on the effects of MDD (overall *N* = 10 105) (Schmaal et al., 2017). However, since this is the largest joint mega-analysis concerning cortical thickness and CM to date, this null finding could also suggest that the effects of MDD commonly reported in studies and meta-analyses may be the result of the interaction of several underlying variables. For example, different effects of MDD depending on age and onset were earlier highlighted in Schmaal et al., (2017). Our findings suggest that CM could be another factor that accounts in part for structural differences between depressed patients and HC.

### Strengths and limitations

A major strength of the study is the large sample size with a relevant control sample allowing inclusion of all 34 left and right cortical brain regions in the analyses. However, a larger sample from different sites also limits the common information collected, since not all sites used the same assessments. Not all patients were drug free and, further, the history of antidepressants use as well as duration, type, and dosage of antidepressant treatment were not assessed completely during the lifetime, so we cannot rule out effects of antidepressant exposure influencing our results. Additionally, psychopathology was assessed with different ratings, so that it was not possible to use depression severity as a single covariate in the analysis. Overall, the datasets from the different samples included in the mega-analysis were significantly heterogeneous regarding demographics and clinical features. This is a common limitation of multi-site analyses and we accounted for this effect by adding site as a covariate to all of our models. It is worth noting that we were able to analyze the influence of clinical confounds in a subset of our MDD cohort, where we did not find any significant effect of recurrence, antidepressant medication, remission, severity or age of depression onset in predicting cortical thickness or surface. However, measures of socioeconomic status and education have been shown to play a role in brain structure (Ritchie et al., 2017), but were unfortunately unavailable in our sample. Finally, we considered hemisphere as a within-subject effect in our dataset. Our hypothesis was that CM would affect anatomically distinct regions differently rather than be selective for a specific region on a specific hemisphere. Therefore, we believed that including all possible interactions between regions and hemisphere would lead to an unnecessarily complex model. However, it is possible that besides the bilateral effects we report, subtler lateralized effects of CM might exist in specific areas.

Even if our investigation features the broad variation of ‘real life’ clinical populations, future studies are needed to confirm our findings in carefully controlled datasets. Here, we explored the effect of CM in a sample of healthy participants and patients with MDD. Because it is not clear how the severity or type of CM may affect the development of structural brain measures, it will be important to consider the onset and timing of CM in future (Ho et al., 2018). In addition, future longitudinal data are required to establish whether cortical thickness might decrease more rapidly with age in individuals with a more severe history of CM, as our current cross-sectional data may suggest. For this analysis, while it was possible to use extracted cortical measures from specific regions of interest, it was not possible to retrospectively analyze the original MRI datasets to perform a whole-cortex analysis with *FreeSurfer*. A surface-based analysis across the entire cortex may afford more sensitivity in detecting effects of CM and thus could be a future step.

## Conclusions

The results of our study support the idea that CM-severity appears to affect the structure of temporal and parietal regions in particular. Thus, there are effects in the default mode network and in regions involved in theory of mind as well as perception. Interestingly, CM may interact with the effect of age on cortical thickness in these regions and others involved in emotion regulation. Thus, future longitudinal studies should investigate if subjects with a history of CM may be more prone to cortical thinning during aging or if CM results in changes that mimic aging.

## Supplementary Material

Supplementary material

## Figures and Tables

**Table 1.** Demographic and clinical data

	All subjects (*n* = 3872)	Controls (*N* = 2588)	Patients (*N* = 1284)	Group difference
Females	2116 (54.6%)	1303 (50.3%)	813 (63.3%)	
Males	1756 (45.4%)	1285 (49.7%)	471 (36.7%)	χ^2^ = 58.2, *p* < 0.001
Age (years)	42.5 ± 15.5	43.3 ± 15.9	40.9 ± 14.6	*t* = 4.6, *p* < 0.001
Age of onset (years)	–	–	29.4 ± 14.0	–
Total CTQ	36.3 ± 12.7	32.6 ± 8.5	43.6 ± 16.1	*p* < 0.001^a^
Sexual abuse	5.5 ± 2.2 (233)	5.2 ± 1.2 (75)	6.2 ± 3.3 (158)	*p* < 0.001^a^
Physical abuse	6.1 ± 2.5 (297)	5.6 ± 1.7 (91)	6.9 ± 3.4 (206)	*p* < 0.001^a^
Emotional abuse	7.6 ± 4.0 (770)	6.5 ± 2.5 (246)	10.0 ± 5.1 (522)	*p* < 0.001^a^
Physical neglect	7.0 ± 2.6 (243)	6.6 ± 2.2 (86)	8.0 ± 3.1 (157)	*p* < 0.001^a^
Emotional neglect	9.9 ± 4.8 (646)	8.6 ± 3.8 (202)	12.5 ± 5.5 (444)	*p* < 0.001^a^
BDI-II^b^	–	5.2 ± 4.4	18.6 ± 12.1	*p* < 0.001^a^
HDRS^b^	–	2.9 ± 3.1	15.6 ± 9.8	*p* < 0.001^a^
ICV (in mm^3^)	(1.53 ± 0.19)×10^6^	(1.54 ± 0.18)×10^6^	(1.527 ± 0.2)×10^6^	*t* = 2.7, *p* = 0.007

CTQ, Childhood Trauma Questionnaire; ICV, total intracranial volume; BDI^#^, Beck Depression Inventory; HDRS-17^#^, Hamilton Depression Rating Scale. Shown are mean values±standard deviation. For CTQ subscales, number of subjects above the cut-off are given in brackets.

a Mann Whitney *U* test used.

b From sites that used these ratings.

**Table 2.** Main findings derived from the GEE models not including the interaction of childhood maltreatment and age

	Participants	Wald χ^2^	*p*_FDR_	Effect
Thickness				
*CM severity*				
Overall thickness	Females	4.861	0.027	−0.001
Rostral anterior cingulate cortex	Males	14.426	<0.001	0.002
Banks of the superior temporal sulcus	All	14.583	0.004	−0.001
Supramarginal gyrus	All	8.889	0.049	−0.001
*CM type*				
Banks of the superior temporal sulcus	Neglect + Abuse > no CM	19.888	0.006	−0.036
Inferior parietal lobe	Neglect + Abuse > no CM	15.273	0.023	−0.022
Middle temporal lobe	Neglect + Abuse > no CM	12.123	0.048	−0.025
Precuneus	Neglect + Abuse > no CM	15.325	0.023	−0.020
Supramarginal gyrus	Neglect + Abuse > no CM	13.990	0.026	−0.024
Surface				
*CM severity*				
Overall surface area	All	4.413	0.036	−0.414
Middle temporal lobe	All	12.368	0.015	−1.504
*CM type*				
Caudal anterior cingulate	Depressed males, Neglect > no CM	17.807	0.003	−44.597
	Depressed males, Abuse > no CM	5.647	0.017	−51.396

Wald χ^2^ and *p* values of CM severity and type are shown for the regions where they were significant. For all effects see Supplemental Tables. Effects are coefficients for the model term CM severity or the estimates of the indicated contrast for CM type. CM, childhood maltreatment; FDR, false discovery rate.

**Table 3.** Main findings derived from the GEE models predicting cortical thickness and including the interaction of childhood maltreatment with age

	Wald χ^2^	*p*_FDR_	Effect
CM severity × Age			
Overall thickness	11.105	0.001	−3.50 × 10^−5^
Banks of superior temporal sulcus	4.997	0.047	−3.59 × 10^−5^
Cuneus	7.373	0.020	−3.32 × 10^−5^
Frontal pole	10.448	0.007	−8.03 × 10^−5^
Fusiform	6.714	0.026	−3.50 × 10^−5^
Insula	8.214	0.014	−4.16 × 10^−5^
Isthmus of cingulate	11.149	0.007	−5.87 × 10^−5^
Lateral orbitofrontal	8.952	0.011	−4.37 × 10^−5^
Parahippocampal	6.031	0.032	−6.00 × 10^−5^
IFG pars opercularis	11.014	0.007	−4.70 × 10^−5^
IFG pars triangularis	8.583	0.011	−4.14 × 10^−5^
Posterior cingulate	17.682	0.001	−5.78 × 10^−5^
Precentral	5.188	0.046	−3.34 × 10^−5^
Precuneus	6.272	0.029	−3.19 × 10^−5^
Rostral anteriorcingulate	10.262	0.007	−5.83 × 10^−5^
Superior frontal	7.301	0.020	−4.22 × 10^−5^
Superior temporal	8.774	0.011	−4.35 × 10^−5^
Supramarginal	5.189	0.046	−3.22 × 10^−5^
Transverse temporal	8.941	0.011	−5.75 × 10^−5^
CM type × Age			
Caudal anterior cingulate	10.155	0.030	−0.002
Caudal middle frontal	16.297	0.002	−0.002
Cuneus	15.442	0.002	−0.001
Frontal pole	16.065	0.002	−0.003
Inferior parietal	9.848	0.034	−0.001
Insula	22.037	<0.001	−0.002
Isthmus of cingulate	23.710	<0.001	−0.003
Lateral orbitofrontal	17.174	0.002	−0.002
Medial orbitofrontal	13.542	0.008	−0.001
Paracentral	9.383	0.039	−0.001
Parahippocampal	16.405	0.002	−0.003
IFG pars opercularis	27.556	<0.001	−0.002
IFG pars orbitalis	8.785	0.047	−0.002
IFG pars triangularis	20.837	<0.001	−0.002
Posterior cingulate	35.357	<0.001	−0.003
Precentral	17.291	0.002	−0.002
Precuneus	13.794	0.006	−0.001
Rostral anteriorcingulate	27.847	<0.001	−0.003
Rostral middlefrontal	9.544	0.037	−0.001
Superior frontal	28.174	<0.001	−0.002
Superior temporal	22.270	<0.001	−0.002
Supramarginal	17.641	0.002	−0.002
Transverse temporal	22.386	<0.001	−0.003

Wald χ^2^ and *p* values of CM severity × Age and CM type × Age are shown for the regions where they were significant. For all effects see Supplemental Tables. Effects are coefficients for the model term CM severity × Age or those for Age in the Neglect + Abuse group *v*. the no-CM group. CM, childhood maltreatment; FDR, false discovery rate.
