# Supplementary material for: Interactive impact of childhood maltreatment, depression, and age on cortical brain structure: mega-analytic findings from a large multi-site cohort
Source: Psychol Med. Author manuscript; Available in PMC 2022 Jul 5. (PMC9254722; doi:10.1017/S003329171900093X)
Supplement: Supplementary material [file NIHMS1814604-supplement-Supplementary_material.docx]

**eTable 1:** **ENIGMA - Major Depressive Disorder Working Group Demographics.** Age (in years), sex, and MDD patients-control breakdown for participating sites. MDD=major depressive disorder.

| Study # | Study name | Age Controls (Mean ± SD) | Age MDD  (Mean ± SD) | % Female Controls | % Female MDD | Total N Controls | Total N MDD | Total N |
| --- | --- | --- | --- | --- | --- | --- | --- | --- |
| 1 | **NESDA** | 40.0 ± 9.7 | 37.6 ± 10.6 | 63.9 | 68 | 61 | 125 | 186 |
| 2 | **Imaging Genetics Dublin** | 36.7 ± 13.1 | 41.6 ± 10.8 | 56.9 | 63.5 | 51 | 52 | 103 |
| 3 | **Clinical Depression Dublin** | 37.7 ± 10.6 | 33.4 ± 8.0 | 52.9 | 33.3 | 17 | 15 | 32 |
| 4 | **CODE** | 38.7 ± 13.2 | 41.2 ± 11.7 | 61.3 | 63.6 | 31 | 88 | 119 |
| 5 | **Münster Neuroimaging** | 36.6 ± 11.8 | 37.4 ± 12.0 | 55.8 | 56 | 547 | 182 | 729 |
| 6 | **SHIP** | 55.3 ± 12.8 | 53.6 ± 11.9 | 44.3 | 71.8 | 420 | 131 | 551 |
| 7 | **SHIP-Trend** | 50.4 ± 14.2 | 49.1 ± 11.9 | 43.7 | 63.9 | 900 | 294 | 1194 |
| 8 | **Sydney** | NA | 18.6 ± 3.0 | NA | 78 | 0 | 50 | 50 |
| 9 | **South Africa** | 28.2 ± 8.5 | NA | 33.9 | NA | 59 | 0 | 59 |
| 10 | **London** | 52.6 ± 5.7 | 49.3 ± 8.0 | 63.2 | 66.7 | 19 | 9 | 28 |
| 11 | **FOR2107-Marburg** | 33.5 ± 12.6 | 37.3 ± 13.6 | 61.5 | 61.3 | 322 | 253 | 575 |
| 12 | **FOR2107-Münster** | 26.2 ± 8.7 | 32.1 ± 13.1 | 65 | 57.5 | 103 | 40 | 143 |
| 13 | **San Francisco** | 15.2 ± 1.4 | 15.6 ± 1.4 | 42.1 | 65.4 | 38 | 26 | 64 |
| 14 | **Magdeburg** | 31.9 ± 9.6 | 39.5 ± 15.6 | 50 | 52.6 | 20 | 19 | 39 |
|  | **Combined** | 43.3 ± 15.9 | 40.9 ± 14.6 | 50.3 | 63.3 | 2588 | 1284 | 3872 |

**eTable 2:** **ENIGMA - Major Depressive Disorder Working Group Clinical characteristics of MDD patients.** Percentage of MDD patients using antidepressant medication, percentage of first episode and recurrent episode MDD patients, percentage of acutely depressed and remitted MDD patients, age of onset of MDD and severity of symptoms breakdown for participating sites. NA: Does not apply. ^a^ Measured with the Hamilton Depression Rating Scale (HDRS-17; range: 0-52), ^b^ Measured with the Beck Depression Inventory (BDI-II; range: 0-63), ^c^ Measured with the Inventory of Depressive Symptomatology-Self report (IDS-SR; range: 0-84), ^d^ BDI-II derived from the PHQ9.

| Study # | Sample | % Antidepressants users | % First episode/recurrent MDD | % Acute/ remitted MDD | Age of onset MDD (mean ± SD) | HDRS-17^a^ Severity MDD (mean ± SD) | BDI-II^b^ Severity MDD (mean ± SD) | IDS-SR^c^ Severity MDD (mean ± SD) |
| --- | --- | --- | --- | --- | --- | --- | --- | --- |
| 1 | **NESDA** | 32.0 | 41.6/58.4 | 100/0 | 23.9 ± 10.5 |  |  | 25.4 ± 12.0 |
| 2 | **Imaging Genetics Dublin** | 71.2 | 15.4/84.6 | 100/0 | 25.3 ± 12.8 | 28.3 ± 6.4 | 33.1 ± 11.7 |  |
| 3 | **Clinical Depression Dublin** | 86.7 | 57.1/42.9 | 100/0 | 30.9 ± 7.1 | 22.7 ± 5.3 |  |  |
| 4 | **CODE** | 0 | 0/100 | 100/0 | NA |  |  | 38.2 ± 11.6 |
| 5 | **Münster Neuroimaging** | 89.9 | 22.0/78.0 | 100/0 | 29.0 ± 12.2 | 23.3 ± 5.1 | 28.0 ± 9.2 |  |
| 6 | **SHIP** | 17.6 | 56.5/43.5 | NA | 38.3 ± 13.2 |  | 11.6 ± 10.2 |  |
| 7 | **SHIP-Trend**^d^ | 17.3 | 37.4/62.6 | NA | 36.0 ± 14.1 |  | 12.4 ± 8.1 |  |
| 8 | **Sydney** | 56 | 18/82 | 28.6/71.4 | 13.8 ± 2.4 | 14.5 ± 6.9 |  |  |
| 9 | **South Africa** | NA | NA | NA | NA |  |  |  |
| 10 | **London** | 77.8 | 0/100 | NA | 21.0 ± 9.1 |  | 16.9 ± 12.7 |  |
| 11 | **FOR2107-Marburg** | 63.1 | 21.3/78.7 | 75.9/24.1 | 26.4 ± 13.0 | 8.2 ± 6.4 | 19.2 ± 11.2 |  |
| 12 | **FOR2107-Münster** | 62.5 | 40.0/60.0 | 72.5/27.5 | 23.5 ± 11.5 | 9.1 ± 7.7 | 15.7 ± 12.0 |  |
| 13 | **San Francisco** | 0 | 34.6 /65.4 | 99.5/0.5 | 12.5 ± 2.7 |  | 30.7 ± 9.7 |  |
| 14 | **Magdeburg** | NA | NA | NA | NA | 17.4 ± 7.2 | 18.8 ± 11.9 |  |

**eTable 3**: **Instrument for diagnosing Major Depressive Disorder and exclusion criteria by site.** MDD: Major Depressive Disorder; CIDI: the Composite International Diagnostic Interview; SCID: Structured Clinical Interview for DSM disorders; SCAN: Schedules for Clinical Assessment in Neuropsychiatry; CESD: Center for Epidemiologic Studies Depression scale; DSM: Diagnostic and Statistical Manual of Mental Disorders; MRI: Magnetic Resonance Imaging; OCD: Obsessive Compulsive Disorder; PTSD: Posttraumatic Stress Disorder.

| Sample | Instrument for diagnosing MDD | Exclusion criteria |
| --- | --- | --- |
| NESDA | CIDI interview | MDD subjects: presence of axis-I disorders other than MDD, panic disorder, social anxiety disorder, or generalized anxiety disorder and any use of psychotropic medication other than stable use of SSRIs or infrequent benzodiazepine use (i.e., equivalent to 2 doses of 10 mg of oxazepam 3 times per week or use within 48 hours prior to scanning).  Control subjects: no Axis-I diagnosis, no medication use.  All subjects: presence or history of major internal or neurological disorder, dependence on or recent abuse (past year) of alcohol and/or drugs, hypertension, and general MRI contraindications. |
| Imaging Genetics Dublin | SCID-1 interview | MDD subjects: comorbid psychiatric disorders (Axis I or Axis II, other than MDD), Treatment with antipsychotics or mood stabilizers, age <18 or >65,  Control subjects: no Axis-I diagnosis, no medication use.  All subjects: history of neurological or other severe medical illness, head injury or severe substance abuse in their lifetime history and general MRI contraindications. |
| Clinical Depression Dublin | SCID-1 interview | MDD subjects: comorbid psychiatric disorders (Axis I or Axis II, other than MDD), Treatment with antipsychotics or mood stabilizers, age <18 or >65,  Control subjects: no Axis-I diagnosis, no medication use.  All subjects: history of neurological or other severe medical illness, head injury or severe substance abuse in their lifetime history and general MRI contraindications. |
| CODE | SCID interview | MDD: Presence of any other Axis-1 diagnosis; Acute risk for suicide (in contrast to suicidal ideation); History of psychotic symptoms, bipolar disorder, or dementia; Schizotypal, antisocial or borderline personality disorder; Use of psychotropic medication within two weeks prior to the start of the study; No current psychotherapeutic treatment.  Control subjects: No history of or current Axis-1 or 2 disorders.  All subjects: History of or current neurological disorder or brain injury; Serious medical condition; Severe cognitive impairment; Substance-related abuse or dependence disorder; Use of psychotropic medication; Use of central-acting medication; Pregnancy; General MRI contraindications. |
| Münster Neuroimaging |  | Inclusion criteria: age 17-65 years; Exclusion criteria all: any MRI contraindications; Exclusion criteria controls: any current or former psychiatric disorder; Exclusion criteria patients: any neurological abnormalities, substance-related disorders or current benzodiazepine treatment (wash out of at least three half-lives before study participation), and former electroconvulsive therapy, bipolar disorder |
| SHIP | M-CIDI interview | MDD subjects: presence of axis-I disorders other than MDD, anxiety disorders, conversion, somatization and eating disorder.  Control subjects: no lifetime diagnosis of depression, no antidepressivants, and severity index=0  All subjects: We removed subjects with medical conditions (e.g. a history of cerebral tumor, stroke, Parkinson’s diseases, multiple sclerosis, epilepsy, hydrocephalus, enlarged ventricles, pathological lesions) or due to technical reasons (e.g. severe movement artefacts or inhomogeneity of the magnetic field). |
| SHIP-Trend | M-CIDI interview | MDD subjects: no special exclusion criteria  Control subjects: no lifetime diagnosis of depression, no antidepressivants, and severity index=0  All subjects: We removed subjects with due to medical conditions (e.g. a history of cerebral tumour, stroke, Parkinson’s diseases, multiple sclerosis, epilepsy, hydrocephalus, enlarged ventricles, pathological lesions) or due to technical reasons (e.g. severe movement artefacts or inhomogeneity of the magnetic field). |
| Sydney | SCID interview | MDD subjects: presence of axis-I disorders other than MDD, panic disorder, social anxiety disorder, or generalized anxiety disorder.  Control subjects: no Axis-I diagnosis, no medication use.  Exclusion criteria for all subjects included medical instability (as determined by a psychiatrist), history of neurological disease (e.g. tumour, head trauma, epilepsy), medical illness known to impact cognitive and brain function (e.g. cancer), intellectual and/or developmental disability and insufficient English for neuropsychological assessment. All subjects were asked to abstain from drug or alcohol use for 48 hours prior to testing and informed about a drug screen protocol. |
| South Africa |  | For controls: current or past Axis-I diagnosis, medical or neurological illness (including renal, hepatic, pulmonary, endocrine disease, encephalitis, epilepsy) or head trauma, severe substance abuse or dependencies other than nicotine, use of central-acting medication, a seropositive test for HIV, MRI incompatibilities or known claustrophobia, insufficient English |
| London | Schedules  for Clinical Assessment in Neuropsychiatry (SCAN) | MDD subjects: Less than two depressive episodes of at least moderate severity. Did not meet DSM-IV diagnostic criteria for recurrent major depressive disorder  Control group participants were clinically interviewed to ensure they had never experienced depressive symptoms.  Exclusion criteria for all participants were for contraindications to MRI; other exclusion criteria were a diagnosis of neurological disorder, head injury leading to loss of consciousness or conditions known to affect brain structure or function (including alcohol or substance misuse), ascertained during clinical interview. Potential participants were also excluded if they or a first-degree relative had ever fulfilled criteria for mania, hypomania, schizophrenia or mood-incongruent psychosis. |
| FOR2107-Marburg | SCID interview | MDD subjects: presence of axis-I disorders other than MDD, Dysthymia and cyclothymia are not inclusion criteria without the presence of MDD, but are allowed as comorbidity.  Controls: No CTQ-subscale reaching the threshold for maltreatment, no family history of any psychiatric disorder, no personal history of any axis I or II disorder in the SCID-I and –II, no current psychotropic medication  Exclusion criteria for all participants: no MRI contraindications (metal implants, pacemakers, claustrophobia, pregnancy, etc.), no history of neurological disorders (e.g., seizures, stroke, multiple sclerosis, dementia, head trauma), no (comorbid) diagnosis of substance-related disorders, no history of severe medical disorders (e.g., cancer, auto-immune disorders, chronic inflammatory diseases, cardio-vascular diseases), Verbal IQ > 80, West-European ancestry |
| FOR2107-Münster | SCID interview | MDD subjects: presence of axis-I disorders other than MDD, Dysthymia and cyclothymia are not inclusion criteria without the presence of MDD, but are allowed as comorbidity.  Controls: No CTQ-subscale reaching the threshold for maltreatment, no family history of any psychiatric disorder, no personal history of any axis I or II disorder in the SCID-I and –II, no current psychotropic medication  Exclusion criteria for all participants: no MRI contraindications (metal implants, pacemakers, claustrophobia, pregnancy, etc.), no history of neurological disorders (e.g., seizures, stroke, multiple sclerosis, dementia, head trauma), no (comorbid) diagnosis of substance-related disorders, no history of severe medical disorders (e.g., cancer, auto-immune disorders, chronic inflammatory diseases, cardio-vascular diseases), Verbal IQ > 80, West-European ancestry |
| San Francisco | All potentially depressed adolescents were administered the Schedule for Affective Disorders and Schizophrenia for School-Age Children-Present and Lifetime Version (Kaufman et al., 1997). At baseline, healthy control participants were administered the Diagnostic Interview Schedule for Children (Shaffer et al., 2000) and Diagnostic Predictive Scale (Lucas et al., 2001) instruments to screen for the presence of DSM-IV-TR Axis I disorders. | The following exclusion criteria were adopted for both healthy and MDD participants:   1. Misuse of drugs (illicit or prescription) within the two months prior to scanning as determined by the CDDR. Two or more alcoholic drinks per week or within the previous month as determined by the CDDR. Urine toxicology screening was not performed. 2. The presence of color blindness determined by the Ishihara Color Plates test. 3. Less than 20/40 correctible vision determined by the standard Snellen Eye Chart. 4. A verbal IQ less than 80 and a performance IQ less than 80 as determined by the WASI. 5. Contraindications for MRI (e.g., ferrometallic implants, braces, or claustrophobia.) 6. Pregnancy or the possibility thereof. 7. Left-handedness. 8. Prepubertal status (Tanner stages 1 or 2.) 9. Inability to understand and comply with study procedures. 10. Use of medications within the 2 weeks before scanning that are known to affect the central nervous system. 11. A history of neurological disorder (e.g., meningitis, migraine), head trauma, a learning disability, serious health problems, a complicated or premature birth before 33 weeks of gestation (due to possibility of abnormal neurodevelopment.)   Depressed adolescents were subject to the following additional exclusion criterion:   1. A primary psychiatric diagnosis other than MDD.   Healthy control adolescents were subject to the following additional exclusion criteria:   1. A Children’s Depression Rating Scale-Revised (CDRS-R) (Poznanski, 1996) t-score greater than 54. 2. Any family history of mood or psychotic disorders in a first or second degree relative as revealed by the FIGS. 3. Current or lifetime DSM-IV-TR Axis I psychiatric disorders, the presence of which was determined by the Diagnostic Interview Schedule for Children (DISC) (Shaffer et al., 2000) and Diagnostic Predictive Scale (DPS) (Lucas et al., 2001) instruments. |
| Magdeburg | ICD-10 interview | MDD subjects: Exclusion criteria were major medical illness, history of seizures, medication with glutamate modulating drugs (ketamine, riluzole, etc.) or benzodiazepines prior electroconvulsive therapy (ECT) treatments and pregnancy, as well as all contraindications against MRI. Specific psychiatric exclusion criteria consisted of atypical forms of depression, any additional psychiatric disorder, and a history of substance abuse or dependence.  Healthy subjects without any psychiatric, neurological, or medical illness were self-referred from study advertisements. All volunteers completed the mini-international neuropsychiatric interview (MINI) to specifically ensure the absence of any ICD-10 psychiatric disorders. |

**eTable 4: Image acquisition parameters.**

| Sample | Scanner vendor and type | Acquisition parameters | Freesurfer version | Slice orientation | Operating system |
| --- | --- | --- | --- | --- | --- |
| NESDA | 3T Phillips Achieva/Intera | 3D gradient-echo T1-weighted sequence. TR=9 msec; TE=3.5 msec; flip angle 8º. FOV = 256 mm; matrix: 25x62x56; in plane voxel size = 1 mm × 1 mm x 1 mm; 170 slices. | 5.0 | Sagittal | Linux-centos4_x86_64 |
| Imaging Genetics Dublin | 3T Phillips Achieva | A sagittal T1 3D TFE was used to scan all participants. TR=8.5 msec; TE=3.9 msec; FOV = 256 mm. AP: 256 mm. RL: 160 mm; matrix: 256×256. voxel size = 1 mm x 1mm x 1 mm | 5.3 | Sagittal | Mac OS |
| Clinical Depression Dublin | 1.5T Siemens Vision | 3D-MPRAGE T1-weighted sequence. TR=11.6 msec; TE=4.9 msec; FOV=230 mm; matrix 512 x 512. slice thickness: 1.5 mm. | 5.3 | Coronal | Mac OS |
| CODE | 3T Siemens Trio (4 Sites). 3 T Philips Achieva (1 site) | Siemens: T1 mprage. voxel size 1 mm x 1 mm x 1 mm; TR=1900 msec; TE=2.52 msec; Sample 1: 192 slices. Sample 2: 176 slices (except 1 site: 192)  Philips: T1 3D-TFE. voxel size 1 mm x 1 mm x 1 mm; TR=8.3 msec; TE=3.8 msec; 170 slices. | 5.3 | Sagittal | Ubuntu 12.04 LTS (Linux 64bit) |
| Münster Neuroimaging | Gyroscan Intera 3T. Philips Medical Systems | 3D fast gradient echo sequence (turbo field echo). TR = 7.4 msec. TE = 3.4 msec. Flip Angle = 9°. two signal averages. inversion prepulse every 814.5 msec. acquired over a FOV of 256 (feet-head [FH]) × 204 (anterior-posterior [AP]) × 160 (right-left [RL]) mm. phase encoding in AP and RL direction. reconstructed to cubic voxels of .5 × .5 × .5 mm | 5.3 | Sagittal | Red Hat Enterprise Linux Server release 5.11 (Tikanga), Linux-centos6_x86_64 |
| SHIP | 1.5T Siemens Avanto | 3D T1-weighted (MP-RAGE/ axial plane); TR=1900 msec; TE=3.4 msec; Flip angle=15°; voxel size 1 mm x 1 mm x 1 mm | 5.3 | Axial | Centos6_x86_64 |
| SHIP-Trend | 1.5T Siemens Avanto | 3D T1-weighted (MP-RAGE/ axial plane); TR=1900 msec; TE=3.4 msec; Flip angle=15°; voxel size 1 mm x 1 mm x 1 mm | 5.3 | Axial | Centos6_x86_64 |
| Sydney | 3T GE MR750 | 3D T1-weighted sequence. TR=7.2 msec; TE=2.78 msec; matrix =256; FOV=240; No. slices=196; thick=0.9mm; inplane resolution=0.9375 | 5.1 | Coronal | Linux_Ubuntu12.04_64 |
| South Africa | 3T Siemens MAGNETOM Allegra | T1-weighted. 3D-MEMPRAGE sequence; TR=2530ms; graded TE=1.53. 3.21. 4.89. 6.57ms; flip angle=7°; FOV=256mm. voxel size=1x1x1mm; 160 slices | 5.3 | Sagittal | Linux-centos4_x86_64 |
| London | 1.5T GE Signa HDx | T1-weighted scans. custom-written pulse sequence. Magnetisation-Prepared Rapid Gradient Echo (MP-RAGE) TE = 3.8 ms. TR = 8.59 ms. flip angle = 8°. field of view = 24 × 24 cm. slice thickness = 1.2 mm. number of slices = 180 and image matrix = 256 × 256. | 5.3 | Sagittal | Linux-centos4_x86_64 |
| FOR2107-Marburg | 3T Tim Trio | 3D MPRAGE sequence: echo time (TE) 2.26ms, repetition time (TR) 1900ms, field of view (FoV) 256mm, matrix 256x256, slice thickness (ST) 1mm, phase encoding direction Anterior >> Posterior, flip angle 9 degree, parallel imaging GRAPPA with acceleration factor 2, bandwidth 200Hz/Px, sagittal acquisition, 176 slices. Total acquisition time is 258 s | 5.3 | Sagittal | Linux-centos6_x86_64 |
| FOR2107-Münster | 3 T Siemens Prisma | 3D MPRAGE sequence: echo time (TE) 2.28ms, repetition time (TR) 2130ms, field of view (FoV) 256mm, matrix 256x256, slice thickness (ST) 1mm, phase encoding direction Anterior >> Posterior, flip angle 8 degree, parallel imaging GRAPPA with acceleration factor 2, bandwidth 200Hz/Px, 176 slices. | 5.3 | Sagittal | Linux-centos6_x86_64 |
| San Francisco | 3T GE MR750 MRI system at UC San Diego | TR/TE=8.1ms/3.17ms. flip angle=12°. 256×256 matrix. 1×1×1mm voxels. 168 sagittal slices | 5.3 | Sagittal | Linux-centos6_x86_64 |
| Magdeburg | 7T Siemens | T1-weighted, 3D-MPRAGE sequence (TE = 2.73 ms, TR = 2300 ms, T1 = 1050 ms, flip angle = 7°, bandwidth = 140 Hz/pixel, acquisition matrix = 320 × 320 x 224, isometric voxel size = 0.8 mm3). | 5.3 | Sagittal | Linux-centos6_x86_64 |

**eTable 5: CM and Clinical Ratings divided by centre.** Demographic and clinical Data. F=females. M=males. CTQ = Childhood trauma questionnaire. ICV= total intracranial volume. BDI= Beck Depression Inventory. HDRS= Hamilton Depression Rating Scale. SA= sexual abuse, PA=physical abuse, EA=emotional abuse, PN=physical neglect, EN=emotional neglect. Shown are mean values +- standard deviation. NA= not available, for NESDA and Clinical Depression Dublin, CTQ was not obtained at the time of scanning, but done retrospectively.

| Sample |  | N | F/M | Age | Onset | CM | SA | PA | EA | PN | EN | BDI-II | HDRS | | ICV |
| --- | --- | --- | --- | --- | --- | --- | --- | --- | --- | --- | --- | --- | --- | --- | --- |
| NESDA | All | 186 | 124/62 | 38.34±10.37 |  | 40.15±13.92 | 5.77±2.62 | 6.04±2.51 | 8.67±4.24 | 7.40±2.88 | 12.26±5.42 | NA | NA | | (1.50+0.18)*10^6^ |
|  | HC | 61 | 39/22 | 39.95±9.73 |  | 33.54±10.59 | 5.38±1.34 | 5.51±2.04 | 6.67±2.76 | 6.52±2.52 | 9.46±4.37 | NA | | NA | (1.48±0.20)*10^6^ |
|  | MDD | 125 | 85/40 | 37.55±10.62 | 23.90±10.50 | 43.37±14.25 | 5.96±3.04 | 6.30±2.68 | 9.64±4.50 | 7.83±2.95 | 13.63±5.37 | NA | NA | | (1.51±0.18)*10^6^ |
| Imaging Genetics Dublin | All | 103 | 62/41 | 39.18±12.20 |  | 39.23±16.86 | 6.87±4.43 | 7.08±4.03 | 8.44±4.65 | 7.31±2.97 | 9.53±4.85 | 18.2±17.5 | 15.7±13.7 | | (1.46+0.24)*10^6^ |
|  | HC | 51 | 29/22 | 36.69±13.13 |  | 31.55±6.51 | 5.49±1.27 | 5.76±1.58 | 6.65±2.68 | 6.25±1.67 | 7.39±2.98 | 3.0 ± 4.5 | 2.9 ±3.2 | | (1.49±0.21)*10^6^ |
|  | MDD | 52 | 33/19 | 41.63±10.78 | 25.33±12.78 | 46.77±20.25 | 8.23±5.82 | 8.37±5.16 | 10.19±5.46 | 8.35±3.56 | 11.63±5.41 | 33.1±11.70 | 28.3±6.4 | | (1.42±0.27)*10^6^ |
| Clinical Depression Dublin | All | 32 | 14/18 | 35.69±9.55 |  | 42.94±18.37 | 5.91±3.60 | 7.09±4.30 | 10.03±5.79 | 7.75±3.42 | 12.16±5.15 | NA | NA | | (1.58±0.14)*10^6^ |
|  | HC | 17 | 9/8 | 37.71±10.59 |  | 41.29±16.69 | 5.47±1.18 | 7.41±3.83 | 9.65±6.27 | 7.18±2.79 | 11.59±5.88 | NA | NA | | (1.54±0.17)*10^6^ |
|  | MDD | 15 | 5/10 | 33.40±7.95 | 30.87±7.09 | 44.80±20.53 | 6.40±5.15 | 6.73±4.89 | 10.47±5.37 | 8.40±4.01 | 12.80±4.28 | NA | 22.7 ± 5.3 | | (1.61±0.10)*10^6^ |
| CODE | All | 119 | 75/44 | 40.55±12.12 |  | 45.24±16.67 | 6.22±3.03 | 6.98±3.81 | 10.87±5.44 | 7.72±2.82 | 13.45±6.02 | NA | NA | | (1.54±0.16)*10^6^ |
|  | HC | 31 | 19/12 | 38.74±13.17 |  | 30.52±6.89 | 5.10±.54 | 5.16±.58 | 6.29±2.04 | 6.13±1.89 | 7.84±3.17 | NA | NA | | (1.55±0.19)*10^6^ |
|  | MDD | 88 | 56/32 | 41.18±11.74 | NA | 50.43±16.00 | 6.61±3.43 | 7.62±4.24 | 12.48±5.35 | 8.28±2.88 | 15.43±5.53 | NA | NA | | (1.53±0.15)*10^6^ |
| Münster Neuroimaging | All | 729 | 407/322 | 36.76±11.87 |  | 36.61±12.57 | 5.54±2.26 | 6.11±2.63 | 8.08±4.06 | 6.82±2.45 | 10.04±4.59 | NA | NA | | (1.44±0.20)*10^6^ |
|  | HC | 547 | 305/242 | 36.56±11.82 |  | 33.50±8.61 | 5.26±1.10 | 5.67±1.78 | 7.10±2.89 | 6.44±2.09 | 9.02±3.76 | NA | NA | | (1.45±0.20)*10^6^ |
|  | MDD | 182 | 102/80 | 37.35±12.04 | 29.03±12.19 | 45.95±17.16 | 6.40±3.99 | 7.44±4.00 | 11.02±5.44 | 7.96±3.04 | 13.12±5.44 | 28.0±9.2 | 23.3±5.1 | | (1.43±0.20)*10^6^ |
| SHIP | All | 551 | 280/271 | 54.91±12.59 |  | 33.35±8.52 | 5.13±.77 | 5.77±1.53 | 6.34±2.31 | 7.09±2.46 | 9.01±4.08 | 5.7±7.3 | NA | | (1.58±0.17)*10^6^ |
|  | HC | 420 | 186/234 | 55.33±12.80 |  | 32.19±7.39 | 5.08±.53 | 5.61±1.30 | 6.03±1.82 | 6.95±2.42 | 8.53±3.70 | 3.8 ± 4.9 | NA | | (1.58±0.17)*10^6^ |
|  | MDD | 131 | 94/37 | 53.56±11.87 | 38.34±13.25 | 37.06±10.63 | 5.29±1.27 | 6.28±2.01 | 7.36±3.23 | 7.55±2.55 | 10.58±4.79 | 11.6±10.2 | NA | | (1.59±0.16)*10^6^ |
| SHIP-Trend | All | 119 | 581/613 | 50.10±13.70 |  | 32.72±9.47 | 5.21±1.22 | 5.66±1.88 | 6.20±2.48 | 6.84±2.47 | 8.81±4.24 | 7.5±5.8 | NA | | (1.60±0.16)*10^6^ |
|  | HC | 900 | 393/507 | 50.44±14.22 |  | 31.39±7.12 | 5.12±.86 | 5.47±1.43 | 5.77±1.59 | 6.65±2.21 | 8.39±3.84 | 6.0 ± 3.7 | NA | | (1.59±0.15)*10^6^ |
|  | MDD | 294 | 188/106 | 49.06±11.94 | 36.02±14.15 | 36.78±13.68 | 5.50±1.93 | 6.23±2.78 | 7.49±3.86 | 7.44±3.06 | 10.12±5.07 | 12.4±8.1 | NA | | (1.61±0.17)*10^6^ |
| Sydney | All | 50 | 39/11 | 18.56±2.98 |  | 45.32±14.98 | 5.94±3.97 | 7.84±3.86 | 11.88±5.38 | 7.54±2.87 | 12.12±5.47 | NA | 14.5±6.9 | | (1.48±0.14)*10^6^ |
|  | HC | 0 |  |  |  |  |  |  |  |  |  |  |  | |  |
|  | MDD | 50 | 39/11 | 18.56±2.98 | 13.82±2.45 | 45.32±14.98 | 5.94±3.97 | 7.84±3.86 | 11.88±5.38 | 7.54±2.87 | 12.12±5.47 | NA | 14.5±6.9 | | (1.48±0.15)*10^6^ |
| South Africa | All | 59 | 20/39 | 28.24±8.49 |  | 35.29±9.93 | 5.98±3.16 | 7.02±3.06 | 7.49±3.38 | 6.32±2.00 | 8.47±3.89 | 6.6±6.8 | NA | | (1.58±0.15)*10^6^ |
|  | HC | 59 | 20/39 | 28.24±8.49 |  | 35.29±9.93 | 5.98±3.16 | 7.02±3.06 | 7.49±3.38 | 6.32±2.00 | 8.47±3.89 | 6.6±6.8 | NA | | (1.58±0.15)*10^6^ |
|  | MDD | 0 |  |  |  |  |  |  |  |  |  |  |  | |  |
| London | All | 28 | 18/10 | 51.57±6.58 |  | 36.82±13.22 | 5.71±2.90 | 5.71±1.36 | 8.18±3.63 | 7.00±3.31 | 10.21±5.25 | NA | NA | | (1.53±0.16)*10^6^ |
|  | HC | 19 | 12/7 | 52.63±5.70 |  | 34.42±13.20 | 6.05±3.50 | 5.58±1.46 | 7.63±3.53 | 6.16±2.29 | 9.00±4.24 | NA | NA | | (1.51±0.16)*10^6^ |
|  | MDD | 9 | 6/3 | 49.33±8.05 | 21.00±9.08 | 41.89±12.47 | 5.00±.00 | 6.00±1.12 | 9.33±3.77 | 8.78±4.47 | 12.78±6.46 | 16.9±12.7 | NA | | (1.56±0.18)*10^6^ |
| FOR2107-Marburg | All | 575 | 353/222 | 35.15±13.19 |  | 39.65±14.99 | 5.91±2.71 | 6.36±2.80 | 9.29±4.89 | 7.20±2.81 | 10.97±5.24 | NA | NA | | (1.53±0.15)*10^6^ |
|  | HC | 322 | 198/124 | 33.48±12.60 |  | 32.92±9.83 | 5.34±1.73 | 5.65±1.87 | 7.19±3.15 | 6.24±1.93 | 8.50±3.72 | NA | NA | | (1.54±0.15)*10^6^ |
|  | MDD | 253 | 155/98 | 37.29±13.63 | 26.44±13.01 | 48.38±16.01 | 6.66±3.46 | 7.28±3.46 | 12.00±5.39 | 8.44±3.25 | 14.15±5.20 | 19.2±11.2 | 8.2±6.4 | | (1.52±0.15)*10^6^ |
| FOR2107-Münster | All | 143 | 90/53 | 27.82±10.43 |  | 34.09±10.00 | 5.35±1.33 | 5.51±1.41 | 7.54±3.52 | 6.47±2.45 | 9.22±4.30 | NA | NA | | (1.58±0.15)*10^6^ |
|  | HC | 103 | 67/36 | 26.17±8.73 |  | 31.51±7.63 | 5.16±.75 | 5.30±.91 | 6.78±2.94 | 6.05±1.79 | 8.23±3.51 | NA | NA | | (1.58±0.15)*10^6^ |
|  | MDD | 40 | 23/17 | 32.07±13.07 | 23.47±11.52 | 40.73±12.22 | 5.85±2.14 | 6.05±2.17 | 9.50±4.12 | 7.55±3.43 | 11.78±5.10 | 15.7±12.0 | 9.1±7.7 | | (1.57±0.15)*10^6^ |
| San Francisco | All | 64 | 33/31 | 15.39±1.41 |  | 51.81±14.50 | 6.59±4.35 | 7.05±3.46 | 10.08±5.38 | 8.05±3.68 | 10.11±4.95 | 14.1±15.5 | NA | | (1.53±0.18)*10^6^ |
|  | HC | 38 | 16/22 | 15.24±1.40 |  | 43.66±5.98 | 5.45±2.09 | 5.66±1.65 | 6.71±2.13 | 6.50±2.46 | 7.29±2.72 | 2.8 ± 4.7 | NA | | (1.58±0.17)*10^6^ |
|  | MDD | 26 | 17/9 | 15.62±1.42 | 12.53±2.74 | 63.73±15.10 | 8.27±6.03 | 9.08±4.35 | 15.00±4.87 | 10.31±4.03 | 14.23±4.60 | 30.7±9.7 | NA | | (1.47±0.17)*10^6^ |
| Magdeburg | All | 39 | 20/19 | 35.59±13.28 |  | 41.62±17.45 | 5.92±3.07 | 6.72±3.87 | 9.36±5.06 | 7.79±3.00 | 11.82±5.46 | NA | NA | | (1.31±0.62)*10^6^ |
|  | HC | 20 | 10/10 | 31.90±9.62 |  | 37.15±17.13 | 5.70±2.70 | 6.70±4.41 | 8.20±4.61 | 7.05±3.07 | 9.50±4.61 | NA | NA | | (1.36±0.62)*10^6^ |
|  | MDD | 19 | 10/9 | 39.47±15.61 | NA | 46.32±16.95 | 6.16±3.48 | 6.74±3.31 | 10.58±5.35 | 8.58±2.80 | 14.26±5.31 | 18.8±11.9 | 17.4±7.2 | | (1.25±0.63)*10^6^ |

e**Table 6: Results of our models predicting cortical thickness based on childhood maltreatment severity (CM and age non interacting).** For brevity, only the effect of interest of each model is shown. For regions featuring a significant interaction, lower level terms were not tested. CM-S=total childhood trauma questionnaire score, FDR=false discovery rate.

| Region | CM-S*Diagnosis*Sex | | Diagnosis*Sex | | Diagnosis*CM-S | | Sex*CM-S | | CM-S | |
| --- | --- | --- | --- | --- | --- | --- | --- | --- | --- | --- |
|  | Wald χ^2^ | p_FDR_ | Wald χ^2^ | p_FDR_ | Wald χ^2^ | p_FDR_ | Wald χ^2^ | p_FDR_ | Wald χ^2^ | p_FDR_ |
| Banks of superior temporal sulcus | 0.023 | 0.935 | 1.702 | 0.768 | 4.250 | 0.223 | 0.164 | 0.777 | 14.583 | 0.004 |
| Caudal anterior cingulate | 1.468 | 0.935 | 0.045 | 0.960 | 0.440 | 0.616 | 1.015 | 0.527 | 1.463 | 0.484 |
| Caudal middle frontal | 0.148 | 0.935 | 0.007 | 0.960 | 4.264 | 0.223 | 0.645 | 0.588 | 1.256 | 0.497 |
| Cuneus | 0.023 | 0.935 | 0.014 | 0.960 | 0.008 | 0.940 | 1.107 | 0.527 | 0.387 | 0.671 |
| Entorhinal cortex | 1.584 | 0.935 | 0.106 | 0.960 | 0.122 | 0.779 | 0.104 | 0.819 | 0.802 | 0.575 |
| Frontal pole | 1.624 | 0.935 | 4.854 | 0.317 | 2.474 | 0.242 | 0.001 | 0.977 | 0.197 | 0.763 |
| Fusiform | 4.503 | 0.935 | 0.637 | 0.768 | 0.594 | 0.555 | 3.441 | 0.242 | 0.757 | 0.575 |
| Inferior parietal | 0.048 | 0.935 | 0.764 | 0.768 | 4.589 | 0.223 | 1.367 | 0.525 | 6.738 | 0.096 |
| Inferior temporal | 1.662 | 0.935 | 0.418 | 0.801 | 3.125 | 0.238 | 6.581 | 0.113 | 3.955 | 0.215 |
| Insula | 0.814 | 0.935 | 0.630 | 0.768 | 2.400 | 0.242 | 4.382 | 0.204 | 0.723 | 0.575 |
| Isthmus of cingulate | 0.008 | 0.956 | 10.288 | 0.034 | 1.185 | 0.408 | 0.908 | 0.527 | 1.783 | 0.182 |
| Lateral occipital | 0.046 | 0.935 | 0.207 | 0.919 | 0.278 | 0.678 | 1.342 | 0.525 | 0.814 | 0.575 |
| Lateral orbitofrontal | 0.125 | 0.935 | 0.032 | 0.960 | 1.610 | 0.317 | 3.778 | 0.242 | 0.062 | 0.878 |
| Lingual | 1.306 | 0.935 | 0.647 | 0.768 | 4.061 | 0.223 | 1.124 | 0.527 | 0.050 | 0.878 |
| Medial orbitofrontal | 0.002 | 0.966 | 0.280 | 0.881 | 0.705 | 0.524 | 2.615 | 0.323 | 0.015 | 0.902 |
| Middle temporal | 0.470 | 0.935 | 0.566 | 0.768 | 2.832 | 0.242 | 2.502 | 0.323 | 5.855 | 0.128 |
| Paracentral | 1.044 | 0.935 | 1.558 | 0.768 | 0.829 | 0.494 | 1.518 | 0.525 | 2.453 | 0.312 |
| Parahippocampal | 0.035 | 0.935 | 1.345 | 0.768 | 1.732 | 0.317 | 0.014 | 0.962 | 2.683 | 0.312 |
| IFG pars opercularis | 0.997 | 0.935 | 1.003 | 0.768 | 5.079 | 0.223 | 0.982 | 0.527 | 3.355 | 0.238 |
| IFG pars orbitalis | 1.075 | 0.935 | 1.315 | 0.768 | 1.043 | 0.435 | 0.937 | 0.527 | 2.066 | 0.372 |
| IFG pars triangularis | 0.082 | 0.935 | 0.122 | 0.960 | 8.025 | 0.170 | 0.618 | 0.588 | 0.367 | 0.671 |
| Pericalcarine | 0.211 | 0.935 | 0.969 | 0.768 | 3.599 | 0.238 | 0.004 | 0.977 | 0.845 | 0.575 |
| Postcentral | 0.066 | 0.935 | 3.461 | 0.490 | 0.006 | 0.940 | 0.353 | 0.695 | 3.983 | 0.215 |
| Posterior cingulate | 0.307 | 0.935 | 5.126 | 0.317 | 1.795 | 0.317 | 0.181 | 0.777 | 1.651 | 0.455 |
| Precentral | 0.403 | 0.935 | 1.041 | 0.768 | 0.360 | 0.644 | 0.572 | 0.588 | 2.570 | 0.312 |
| Precuneus | 0.406 | 0.935 | 0.044 | 0.960 | 3.284 | 0.238 | 7.840 | 0.085 | 3.425 | 0.238 |
| Rostral anteriorcingulate | 0.025 | 0.935 | 0.002 | 0.964 | 1.633 | 0.317 | 13.556 | 0.008 |  |  |
| Rostral middlefrontal | 0.264 | 0.935 | 0.012 | 0.960 | 2.566 | 0.242 | 1.423 | 0.525 | 1.250 | 0.497 |
| Superior frontal | 0.033 | 0.935 | 0.586 | 0.768 | 2.429 | 0.242 | 2.868 | 0.306 | 0.184 | 0.763 |
| Superior parietal | 0.367 | 0.935 | 0.483 | 0.788 | 1.895 | 0.317 | 3.588 | 0.242 | 4.882 | 0.173 |
| Superior temporal | 0.187 | 0.935 | 0.060 | 0.960 | 3.285 | 0.238 | 4.698 | 0.204 | 0.451 | 0.669 |
| Supramarginal | 0.485 | 0.935 | 1.949 | 0.768 | 3.984 | 0.223 | 0.791 | 0.553 | 8.889 | 0.048 |
| Temporal pole | 1.579 | 0.935 | 3.242 | 0.490 | 0.116 | 0.779 | 4.840 | 0.204 | 0.033 | 0.883 |
| Transverse temporal | 2.244 | 0.935 | 2.441 | 0.669 | 2.401 | 0.242 | 0.216 | 0.777 | 0.563 | 0.630 |

**eTable 7: Results of our models predicting cortical thickness based on childhood maltreatment severity (CM-S and age interacting).** For brevity, only the effect of interest of each model is shown. For regions featuring a significant interaction, lower level terms were not tested. CM-S=total childhood trauma questionnaire score, FDR=false discovery rate.

| Region | CM-S*Age | |
| --- | --- | --- |
|  | Wald χ^2^ | p_FDR_ |
| Banks of superior temporal sulcus | 4.997 | 0.047 |
| Caudal anterior cingulate | 3.682 | 0.085 |
| Caudal middle frontal | 3.982 | 0.078 |
| Cuneus | 7.373 | 0.020 |
| Entorhinal cortex | 1.746 | 0.211 |
| Frontal pole | 10.448 | 0.007 |
| Fusiform | 6.714 | 0.026 |
| Inferior parietal | 1.885 | 0.199 |
| Inferior temporal | 1.538 | 0.236 |
| Insula | 8.214 | 0.014 |
| Isthmus of cingulate | 11.149 | 0.007 |
| Lateral occipital | 3.063 | 0.109 |
| Lateral orbitofrontal | 8.952 | 0.011 |
| Lingual | 1.891 | 0.199 |
| Medial orbitofrontal | 3.766 | 0.084 |
| Middle temporal | 3.130 | 0.109 |
| Paracentral | 2.391 | 0.160 |
| Parahippocampal | 6.031 | 0.032 |
| IFG pars opercularis | 11.014 | 0.007 |
| IFG pars orbitalis | 4.380 | 0.064 |
| IFG pars triangularis | 8.583 | 0.011 |
| Pericalcarine | 1.918 | 0.199 |
| Postcentral | 0.220 | 0.658 |
| Posterior cingulate | 17.682 | 0.001 |
| Precentral | 5.188 | 0.046 |
| Precuneus | 6.272 | 0.029 |
| Rostral anteriorcingulate | 10.262 | 0.007 |
| Rostral middlefrontal | 3.073 | 0.109 |
| Superior frontal | 7.301 | 0.020 |
| Superior parietal | 1.023 | 0.332 |
| Superior temporal | 8.774 | 0.011 |
| Supramarginal | 5.189 | 0.046 |
| Temporal pole | 0.001 | 0.978 |
| Transverse temporal | 8.941 | 0.011 |

**eTable 8: Results of our models predicting cortical surface based on childhood maltreatment severity (CM-S and age non interacting).** For brevity, only the effect of interest of each model is shown. For regions featuring a significant interaction, lower level terms were not tested. CM-S=total childhood trauma questionnaire score, FDR=false discovery rate.

| Region | CM-S*Diagnosis*Sex | | Diagnosis*Sex | | Diagnosis*CM-S | | Sex*CM-S | | CM-S |  |
| --- | --- | --- | --- | --- | --- | --- | --- | --- | --- | --- |
|  | Wald χ^2^ | p_FDR_ | Wald χ^2^ | p_FDR_ | Wald χ^2^ | p_FDR_ | Wald χ^2^ | p_FDR_ | Wald χ^2^ | p_FDR_ |
| Banks of superior temporal sulcus | 0.010 | 0.969 | 2.795 | 0.227 | 0.077 | 0.917 | 0.457 | 0.606 | 0.252 | 0.777 |
| Caudal anterior cingulate | 3.902 | 0.449 | 5.019 | 0.180 | 0.051 | 0.918 | 1.188 | 0.469 | 0.019 | 0.892 |
| Caudal middle frontal | 0.599 | 0.711 | 3.002 | 0.227 | 0.105 | 0.917 | 0.981 | 0.498 | 1.703 | 0.363 |
| Cuneus | 0.015 | 0.969 | 0.076 | 0.829 | 0.195 | 0.917 | 2.746 | 0.306 | 1.447 | 0.389 |
| Entorhinal cortex | 1.459 | 0.711 | 0.498 | 0.609 | 0.027 | 0.918 | 2.487 | 0.306 | 0.056 | 0.839 |
| Frontal pole | 1.089 | 0.711 | 2.764 | 0.227 | 0.983 | 0.917 | 2.132 | 0.306 | 0.173 | 0.793 |
| Fusiform | 0.931 | 0.711 | 0.997 | 0.541 | 0.747 | 0.917 | 4.859 | 0.281 | 1.588 | 0.372 |
| Inferior parietal | 0.601 | 0.711 | 4.499 | 0.180 | 0.257 | 0.917 | 0.718 | 0.540 | 0.057 | 0.839 |
| Inferior temporal | 0.116 | 0.946 | 3.558 | 0.223 | 0.401 | 0.917 | 6.076 | 0.238 | 3.213 | 0.209 |
| Insula | 0.716 | 0.711 | 7.501 | 0.180 | 0.795 | 0.917 | 1.189 | 0.469 | 5.443 | 0.150 |
| Isthmus of cingulate | 0.546 | 0.711 | 5.381 | 0.180 | 3.407 | 0.737 | 3.449 | 0.306 | 4.311 | 0.185 |
| Lateral occipital | 0.002 | 0.969 | 4.372 | 0.180 | 0.080 | 0.917 | 2.346 | 0.306 | 0.244 | 0.777 |
| Lateral orbitofrontal | 0.612 | 0.711 | 0.750 | 0.571 | 0.198 | 0.917 | 1.759 | 0.370 | 3.095 | 0.209 |
| Lingual | 1.308 | 0.711 | 0.284 | 0.696 | 0.305 | 0.917 | 0.307 | 0.680 | 2.950 | 0.209 |
| Medial orbitofrontal | 2.097 | 0.689 | 0.061 | 0.829 | 0.019 | 0.918 | 0.623 | 0.549 | 0.844 | 0.507 |
| Middle temporal | 0.065 | 0.969 | 3.235 | 0.227 | 0.331 | 0.917 | 2.326 | 0.306 | 12.368 | 0.015 |
| Paracentral | 0.876 | 0.711 | 1.981 | 0.318 | 1.881 | 0.917 | 0.079 | 0.827 | 1.212 | 0.439 |
| Parahippocampal | 2.597 | 0.606 | 2.840 | 0.227 | 0.242 | 0.917 | 2.449 | 0.306 | 0.391 | 0.724 |
| IFG pars opercularis | 0.775 | 0.711 | 0.849 | 0.555 | 0.100 | 0.917 | 0.862 | 0.510 | 0.219 | 0.777 |
| IFG pars orbitalis | 0.110 | 0.946 | 0.000 | 0.987 | 3.401 | 0.737 | 3.302 | 0.306 | 0.967 | 0.504 |
| IFG pars triangularis | 0.010 | 0.969 | 0.490 | 0.609 | 0.198 | 0.917 | 7.755 | 0.170 | 3.123 | 0.209 |
| Pericalcarine | 1.125 | 0.711 | 0.340 | 0.680 | 0.001 | 0.973 | 0.022 | 0.883 | 1.899 | 0.336 |
| Postcentral | 0.595 | 0.711 | 0.842 | 0.555 | 0.445 | 0.917 | 0.101 | 0.824 | 5.263 | 0.150 |
| Posterior cingulate | 4.700 | 0.449 | 4.421 | 0.180 | 0.083 | 0.917 | 3.574 | 0.306 | 0.149 | 0.793 |
| Precentral | 0.425 | 0.760 | 3.623 | 0.223 | 0.336 | 0.917 | 0.837 | 0.510 | 3.025 | 0.209 |
| Precuneus | 1.437 | 0.711 | 0.996 | 0.541 | 0.189 | 0.917 | 1.061 | 0.491 | 0.057 | 0.839 |
| Rostral anteriorcingulate | 5.246 | 0.449 | 2.702 | 0.227 | 0.246 | 0.917 | 0.165 | 0.776 | 5.670 | 0.150 |
| Rostral middlefrontal | 0.101 | 0.946 | 5.642 | 0.180 | 1.478 | 0.917 | 4.567 | 0.281 | 3.531 | 0.209 |
| Superior frontal | 3.377 | 0.449 | 0.680 | 0.579 | 0.386 | 0.917 | 0.052 | 0.844 | 2.501 | 0.242 |
| Superior parietal | 1.952 | 0.689 | 0.506 | 0.609 | 3.517 | 0.737 | 0.607 | 0.549 | 2.609 | 0.240 |
| Superior temporal | 0.002 | 0.969 | 2.197 | 0.293 | 0.021 | 0.918 | 2.142 | 0.306 | 5.252 | 0.150 |
| Supramarginal | 3.597 | 0.449 | 1.856 | 0.327 | 0.407 | 0.917 | 2.663 | 0.306 | 3.536 | 0.209 |
| Temporal pole | 0.299 | 0.827 | 0.224 | 0.721 | 0.200 | 0.917 | 1.201 | 0.469 | 4.740 | 0.164 |
| Transverse temporal | 0.007 | 0.969 | 0.187 | 0.729 | 0.127 | 0.917 | 2.320 | 0.306 | 0.889 | 0.507 |

**eTable 9: Results of our models predicting cortical surface based on childhood maltreatment severity (CM-S and age interacting).** For brevity, only the effect of interest of each model is shown. For regions featuring a significant interaction, lower level terms were not tested. CM-S=total childhood trauma questionnaire score, FDR=false discovery rate.

| Region | Age * CM-S | |
| --- | --- | --- |
|  | Wald χ^2^ | p_FDR_ |
| Banks of superior temporal sulcus | 0.333 | 0.856 |
| Caudal anterior cingulate | 0.505 | 0.856 |
| Caudal middle frontal | 0.533 | 0.856 |
| Cuneus | 0.125 | 0.945 |
| Entorhinal cortex | 0.307 | 0.856 |
| Frontal pole | 1.651 | 0.856 |
| Fusiform | 7.768 | 0.170 |
| Inferior parietal | 3.664 | 0.759 |
| Inferior temporal | 2.312 | 0.856 |
| Insula | 0.061 | 0.950 |
| Isthmus of cingulate | <0.001 | 0.996 |
| Lateral occipital | 2.517 | 0.856 |
| Lateral orbitofrontal | 0.941 | 0.856 |
| Lingual | 0.001 | 0.996 |
| Medial orbitofrontal | 0.798 | 0.856 |
| Middle temporal | 0.167 | 0.945 |
| Paracentral | 1.084 | 0.856 |
| Parahippocampal | 0.093 | 0.950 |
| IFG pars opercularis | <0.001 | 0.996 |
| IFG pars orbitalis | 1.552 | 0.856 |
| IFG pars triangularis | 0.444 | 0.856 |
| Pericalcarine | 0.729 | 0.856 |
| Postcentral | 0.433 | 0.856 |
| Posterior cingulate | 0.010 | 0.996 |
| Precentral | 0.314 | 0.856 |
| Precuneus | 1.348 | 0.856 |
| Rostral anteriorcingulate | 0.058 | 0.950 |
| Rostral middlefrontal | 3.348 | 0.759 |
| Superior frontal | 0.915 | 0.856 |
| Superior parietal | 0.003 | 0.996 |
| Superior temporal | 0.752 | 0.856 |
| Supramarginal | 0.148 | 0.945 |
| Temporal pole | 1.477 | 0.856 |
| Transverse temporal | 0.565 | 0.856 |

e**Table 10: Results of our models predicting** **cortical thickness based on childhood maltreatment type** **(CM and age non interacting).** For brevity, only the effect of interest of each model is shown. For regions featuring a significant interaction, lower level terms were not tested. CM-T=Childhood maltreatment type (no maltreatment, neglect, abuse, neglect+abuse), FDR=false discovery rate.

| Region | CM-T*Diagnosis*Sex | | Diagnosis*Sex | | Diagnosis*CM-T | | Sex*CM-T | | CM-T | |
| --- | --- | --- | --- | --- | --- | --- | --- | --- | --- | --- |
|  | Wald χ2 | p_FDR_ | Wald χ2 | p_FDR_ | Wald χ2 | p_FDR_ | Wald χ2 | p_FDR_ | Wald χ2 | p_FDR_ |
| Banks of superior temporal sulcus | 7.511 | 0.415 | 1.537 | 0.791 | 1.913 | 0.991 | 0.753 | 0.959 | 19.888 | 0.006 |
| Caudal anterior cingulate | .553 | 0.964 | 0.064 | 0.966 | 0.714 | 0.991 | 0.559 | 0.959 | 2.278 | 0.615 |
| Caudal middle frontal | 4.794 | 0.636 | 0.005 | 0.971 | 0.471 | 0.991 | 0.267 | 0.966 | 6.216 | 0.263 |
| Cuneus | .803 | 0.964 | 0.019 | 0.971 | 1.942 | 0.991 | 2.005 | 0.959 | 6.910 | 0.232 |
| Entorhinal cortex | .152 | 1.000 | 0.133 | 0.964 | 1.478 | 0.991 | 2.523 | 0.959 | 2.234 | 0.615 |
| Frontal pole | 4.800 | 0.636 | 4.757 | 0.329 | 6.644 | 0.991 | 0.780 | 0.959 | 4.740 | 0.363 |
| Fusiform | 7.870 | 0.415 | 0.650 | 0.791 | 1.057 | 0.991 | 1.123 | 0.959 | 4.595 | 0.365 |
| Inferior parietal | .000 | 1.000 | 0.711 | 0.791 | 1.899 | 0.991 | 3.570 | 0.959 | 15.273 | 0.023 |
| Inferior temporal | 7.383 | 0.415 | 0.383 | 0.828 | 1.314 | 0.991 | 4.266 | 0.884 | 6.151 | 0.263 |
| Insula | 2.146 | 0.847 | 0.616 | 0.791 | 0.491 | 0.991 | 4.561 | 0.884 | 3.257 | 0.523 |
| Isthmus of cingulate | .591 | 0.964 | 10.165 | 0.034 | 2.515 | 0.991 | 2.203 | 0.959 | 2.018 | 0.615 |
| Lateral occipital | 1.527 | 0.919 | 0.214 | 0.912 | 3.809 | 0.991 | 4.278 | 0.884 | 6.036 | 0.263 |
| Lateral orbitofrontal | 2.965 | 0.759 | 0.026 | 0.971 | 0.360 | 0.991 | 3.639 | 0.959 | 2.841 | 0.567 |
| Lingual | 1.612 | 0.919 | 0.697 | 0.791 | 2.041 | 0.991 | 0.625 | 0.959 | 5.395 | 0.308 |
| Medial orbitofrontal | 2.198 | 0.847 | 0.293 | 0.869 | 2.329 | 0.991 | 2.977 | 0.959 | 1.231 | 0.769 |
| Middle temporal | 5.791 | 0.519 | 0.485 | 0.826 | 0.548 | 0.991 | 1.249 | 0.959 | 12.123 | 0.048 |
| Paracentral | 4.151 | 0.723 | 1.495 | 0.791 | 0.695 | 0.991 | 1.652 | 0.959 | 3.701 | 0.457 |
| Parahippocampal | .669 | 0.964 | 1.339 | 0.791 | 1.754 | 0.991 | 5.044 | 0.884 | 7.007 | 0.232 |
| IFG pars opercularis | 2.933 | 0.759 | 0.944 | 0.791 | 0.672 | 0.991 | 1.486 | 0.959 | 9.929 | 0.097 |
| IFG pars orbitalis | 6.039 | 0.519 | 1.271 | 0.791 | 0.433 | 0.991 | 1.919 | 0.959 | 2.858 | 0.567 |
| IFG pars triangularis | 7.936 | 0.415 | 0.113 | 0.964 | 1.971 | 0.991 | 2.720 | 0.959 | 2.028 | 0.615 |
| Pericalcarine | 1.223 | 0.942 | 1.127 | 0.791 | 1.685 | 0.991 | 0.480 | 0.959 | 9.460 | 0.102 |
| Postcentral | 2.938 | 0.759 | 3.379 | 0.476 | 0.379 | 0.991 | 0.629 | 0.959 | 9.852 | 0.097 |
| Posterior cingulate | 2.118 | 0.847 | 5.054 | 0.329 | 0.744 | 0.991 | 2.180 | 0.959 | 2.464 | 0.615 |
| Precentral | 1.000 | 0.964 | 1.009 | 0.791 | 2.090 | 0.991 | 0.444 | 0.959 | 5.907 | 0.263 |
| Precuneus | 4.056 | 0.723 | .050 | 0.966 | 0.152 | 0.991 | 6.343 | 0.768 | 15.325 | 0.023 |
| Rostral anteriorcingulate | 2.525 | 0.843 | .000 | 0.995 | 1.562 | 0.991 | 10.444 | 0.459 | 1.968 | 0.615 |
| Rostral middlefrontal | 5.926 | 0.519 | .010 | 0.971 | 0.834 | 0.991 | 1.817 | 0.959 | 4.915 | 0.356 |
| Superior frontal | 3.055 | 0.759 | .590 | 0.791 | 0.107 | 0.991 | 3.032 | 0.959 | 2.154 | 0.615 |
| Superior parietal | 1.773 | 0.918 | .416 | 0.828 | 1.707 | 0.991 | 5.982 | 0.768 | 8.602 | 0.132 |
| Superior temporal | 10.107 | 0.415 | .062 | 0.966 | 1.634 | 0.991 | 9.220 | 0.459 | 3.851 | 0.453 |
| Supramarginal | 3.062 | 0.759 | 1.846 | 0.791 | 2.329 | 0.991 | 2.033 | 0.959 | 13.990 | 0.026 |
| Temporal pole | 1.298 | 0.942 | 3.277 | 0.476 | 0.862 | 0.991 | 6.009 | 0.768 | .549 | 0.908 |
| Transverse temporal | 3.756 | 0.756 | 2.456 | 0.663 | 3.009 | 0.991 | 0.942 | 0.959 | 3.833 | 0.453 |

**eTable 11: Results of our models predicting cortical thickness based on childhood maltreatment type (CM-T and age interacting).** For brevity, only the effect of interest of each model is shown. For regions featuring a significant interaction, lower level terms were not tested. CM-T=Childhood maltreatment type, FDR=false discovery rate.

| Region | CM-T*Age | |
| --- | --- | --- |
|  | Wald χ^2^ | p_FDR_ |
| Banks of superior temporal sulcus | 8.313 | 0.057 |
| Caudal anterior cingulate | 10.155 | 0.030 |
| Caudal middle frontal | 16.297 | 0.002 |
| Cuneus | 15.442 | 0.002 |
| Entorhinal cortex | 1.574 | 0.685 |
| Frontal pole | 16.065 | 0.002 |
| Fusiform | 4.344 | 0.257 |
| Inferior parietal | 9.848 | 0.034 |
| Inferior temporal | 2.034 | 0.600 |
| Insula | 22.037 | <0.001 |
| Isthmus of cingulate | 23.710 | <0.001 |
| Lateral occipital | 4.621 | 0.237 |
| Lateral orbitofrontal | 17.174 | 0.002 |
| Lingual | 3.597 | 0.338 |
| Medial orbitofrontal | 13.542 | 0.008 |
| Middle temporal | 6.543 | 0.115 |
| Paracentral | 9.383 | 0.039 |
| Parahippocampal | 16.405 | 0.002 |
| IFG pars opercularis | 27.556 | <0.001 |
| IFG pars orbitalis | 8.785 | 0.047 |
| IFG pars triangularis | 20.837 | <0.001 |
| Pericalcarine | 4.805 | 0.227 |
| Postcentral | 7.680 | 0.072 |
| Posterior cingulate | 35.357 | <0.001 |
| Precentral | 17.291 | 0.002 |
| Precuneus | 13.794 | 0.006 |
| Rostral anteriorcingulate | 27.847 | <0.001 |
| Rostral middlefrontal | 9.544 | 0.037 |
| Superior frontal | 28.174 | <0.001 |
| Superior parietal | 5.251 | 0.194 |
| Superior temporal | 22.270 | <0.001 |
| Supramarginal | 17.641 | 0.002 |
| Temporal pole | .644 | 0.886 |
| Transverse temporal | 22.386 | <0.001 |

e**Table 12: Results of our models predicting** **cortical surface based on childhood maltreatment type** **(CM and age non interacting).** For brevity, only the effect of interest of each model is shown. For regions featuring a significant interaction, lower level terms were not tested. CM-T=Childhood maltreatment type (no maltreatment, neglect, abuse, neglect+abuse), FDR=false discovery rate.

| Region | CM-T*Diagnosis*Sex | | Diagnosis*Sex | | Diagnosis*CM-T | | Sex*CM-T | | CM-T | |
| --- | --- | --- | --- | --- | --- | --- | --- | --- | --- | --- |
|  | Wald χ2 | p_FDR_ | Wald χ2 | p_FDR_ | Wald χ2 | p_FDR_ | Wald χ2 | p_FDR_ | Wald χ2 | p_FDR_ |
| Banks of superior temporal sulcus | 10.346 | 0.181 | 2.772 | 0.227 | 7.375 | 0.519 | 4.204 | 0.390 | 2.622 | 0.806 |
| Caudal anterior cingulate | 17.807 | <0.001 | 4.971 | 0.175 | 1.070 | 0.961 | 4.196 | 0.390 | 0.116 | 0.990 |
| Caudal middle frontal | 2.287 | 0.796 | 3.142 | 0.227 | 0.550 | 0.965 | 2.039 | 0.661 | 2.156 | 0.820 |
| Cuneus | 1.787 | 0.849 | 0.092 | 0.789 | 0.256 | 0.976 | 6.572 | 0.329 | 1.551 | 0.845 |
| Entorhinal cortex | 0.456 | 1.000 | 0.545 | 0.602 | 11.795 | 0.113 | 7.565 | 0.272 | 2.993 | 0.806 |
| Frontal pole | 3.500 | 0.606 | 2.709 | 0.227 | 4.912 | 0.564 | 5.204 | 0.381 | 0.309 | 0.990 |
| Fusiform | 1.532 | 0.883 | 1.044 | 0.527 | 5.476 | 0.564 | 7.746 | 0.272 | 3.324 | 0.806 |
| Inferior parietal | 8.026 | 0.261 | 4.406 | 0.175 | 3.054 | 0.641 | 2.599 | 0.556 | 2.983 | 0.806 |
| Inferior temporal | 11.564 | 0.153 | 3.62 | 0.214 | 5.057 | 0.564 | 10.297 | 0.272 | 5.206 | 0.667 |
| Insula | 2.025 | 0.838 | 7.786 | 0.170 | 11.352 | 0.113 | 4.997 | 0.384 | 7.063 | 0.667 |
| Isthmus of cingulate | 4.235 | 0.512 | 5.2 | 0.175 | 3.248 | 0.641 | 3.713 | 0.400 | 5.647 | 0.667 |
| Lateral occipital | 1.152 | 0.937 | 4.433 | 0.175 | 2.247 | 0.799 | 7.562 | 0.272 | 0.718 | 0.990 |
| Lateral orbitofrontal | 0.856 | 0.947 | 0.849 | 0.528 | 0.806 | 0.961 | 4.737 | 0.384 | 1.836 | 0.820 |
| Lingual | 4.033 | 0.516 | 0.306 | 0.680 | 1.480 | 0.934 | .060 | 0.996 | 5.555 | 0.667 |
| Medial orbitofrontal | 0.214 | 1.000 | 0.089 | 0.789 | 3.594 | 0.641 | 3.216 | 0.471 | 1.745 | 0.820 |
| Middle temporal | 6.855 | 0.327 | 3.467 | 0.214 | 5.052 | 0.564 | 5.633 | 0.371 | 11.053 | 0.374 |
| Paracentral | 4.453 | 0.512 | 2.069 | 0.300 | 2.970 | 0.641 | 4.184 | 0.390 | 0.564 | 0.990 |
| Parahippocampal | 4.797 | 0.512 | 2.872 | 0.227 | 3.213 | 0.641 | 7.246 | 0.272 | 2.864 | 0.806 |
| IFG pars opercularis | 0.950 | 0.947 | 0.906 | 0.527 | 0.639 | 0.965 | 1.164 | 0.836 | 2.457 | 0.806 |
| IFG pars orbitalis | 1.760 | 0.849 | 0 | 0.998 | 4.690 | 0.564 | 2.612 | 0.556 | 1.235 | 0.905 |
| IFG pars triangularis | 2.632 | 0.732 | 0.547 | 0.602 | 2.040 | 0.799 | 7.490 | 0.272 | 2.060 | 0.820 |
| Pericalcarine | 1.120 | 0.937 | 0.378 | 0.655 | 0.871 | 0.961 | 0.528 | 0.941 | 2.377 | 0.806 |
| Postcentral | 7.246 | 0.311 | 0.917 | 0.527 | 3.126 | 0.641 | 1.415 | 0.796 | 3.970 | 0.806 |
| Posterior cingulate | 8.113 | 0.261 | 4.46 | 0.175 | 3.034 | 0.641 | 6.047 | 0.362 | 0.202 | 0.990 |
| Precentral | 5.610 | 0.449 | 3.774 | 0.214 | .207 | 0.976 | 4.503 | 0.390 | 4.424 | 0.806 |
| Precuneus | <0.001 | 1.000 | 1.029 | 0.527 | 13.005 | 0.113 | 4.022 | 0.390 | 0.297 | 0.990 |
| Rostral anteriorcingulate | 8.016 | 0.261 | 2.868 | 0.227 | 2.077 | 0.799 | 5.437 | 0.371 | 3.679 | 0.806 |
| Rostral middlefrontal | 6.426 | 0.351 | 5.902 | 0.175 | 1.129 | 0.961 | 5.898 | 0.362 | 6.103 | 0.667 |
| Superior frontal | 4.197 | 0.512 | 0.725 | 0.558 | 5.193 | 0.564 | 0.784 | 0.906 | 3.330 | 0.806 |
| Superior parietal | 0.117 | 1.000 | 0.464 | 0.625 | 3.436 | 0.641 | 4.761 | 0.384 | 2.634 | 0.806 |
| Superior temporal | 4.310 | 0.512 | 2.312 | 0.272 | 3.565 | 0.641 | 3.977 | 0.390 | 8.623 | 0.595 |
| Supramarginal | 4.607 | 0.512 | 1.896 | 0.319 | 5.515 | 0.564 | 7.774 | 0.272 | 5.237 | 0.667 |
| Temporal pole | 3.071 | 0.682 | 0.275 | 0.680 | 0.995 | 0.961 | 3.759 | 0.400 | 1.819 | 0.820 |
| Transverse temporal | 2.661 | 0.732 | 0.216 | 0.704 | 4.652 | 0.564 | 12.915 | 0.170 | 3.093 | 0.806 |

**eTable 13: Results of our models predicting cortical surface based on childhood maltreatment type (CM-T and age interacting).** For brevity, only the effect of interest of each model is shown. For regions featuring a significant interaction, lower level terms were not tested. CM-T=Childhood maltreatment type, FDR=false discovery rate.

| Region | CM-T*Age | |
| --- | --- | --- |
|  | Wald χ^2^ | p_FDR_ |
| Banks of superior temporal sulcus | 5.214 | 0.593 |
| Caudal anterior cingulate | 3.096 | 0.712 |
| Caudal middle frontal | 2.137 | 0.775 |
| Cuneus | 2.124 | 0.775 |
| Entorhinal cortex | 0.747 | 0.945 |
| Frontal pole | 3.250 | 0.712 |
| Fusiform | 10.611 | 0.238 |
| Inferior parietal | 3.545 | 0.712 |
| Inferior temporal | 4.497 | 0.649 |
| Insula | 7.434 | 0.401 |
| Isthmus of cingulate | 1.685 | 0.806 |
| Lateral occipital | 5.685 | 0.544 |
| Lateral orbitofrontal | 2.494 | 0.736 |
| Lingual | 6.520 | 0.432 |
| Medial orbitofrontal | 14.939 | 0.068 |
| Middle temporal | 4.318 | 0.649 |
| Paracentral | 0.576 | 0.958 |
| Parahippocampal | 0.315 | 0.974 |
| IFG pars opercularis | 8.529 | 0.306 |
| IFG pars orbitalis | 1.869 | 0.785 |
| IFG pars triangularis | 9.486 | 0.261 |
| Pericalcarine | 7.000 | 0.408 |
| Postcentral | 2.514 | 0.736 |
| Posterior cingulate | 3.239 | 0.712 |
| Precentral | 2.550 | 0.736 |
| Precuneus | 3.583 | 0.712 |
| Rostral anteriorcingulate | 1.268 | 0.895 |
| Rostral middlefrontal | 0.999 | 0.919 |
| Superior frontal | 2.544 | 0.736 |
| Superior parietal | 0.224 | 0.974 |
| Superior temporal | 0.961 | 0.919 |
| Supramarginal | 3.633 | 0.712 |
| Temporal pole | 4.434 | 0.649 |
| Transverse temporal | 1.984 | 0.783 |

**eTable 14: MDD patients with matching clinical information.** Details on age, sex, clinical severity, currently depressed participants (versus remitted), recurrent (versus first episode) and currently taking antidepressants. For this analysis, remission was defined as BDI≤12. MDD: major depressive disorder, BDI: Beck depression inventory.

| Study name | Age  (Mean ± SD) | % Female | Age of onset | BDI | % Current | % Recurrent | % Antidepressant | Total N |
| --- | --- | --- | --- | --- | --- | --- | --- | --- |
| Imaging Genetics Dublin | 41.6 ± 10.8 | 63.5 | 25.3 ± 12.8 | 33.1 ± 11.7 | 96.2 | 15.4 | 73.1 | 52 |
| Münster Neuroimaging | 37.4 ± 12.1 | 55.9 | 29.1 ± 12.2 | 28.1 ± 9.2 | 97.2 | 78.2 | 89.9 | 179 |
| SHIP | 49.1 ± 11.9 | 63.9 | 36 ± 14.1 | 12.4 ± 8.1 | 41.5 | 62.6 | 17.3 | 294 |
| SHIP-Trend | 53.6 ± 11.9 | 71.8 | 38.3 ± 13.2 | 11.6 ± 10.2 | 38.9 | 43.5 | 17.6 | 131 |
| London | 51.4 ± 5.6 | 75 | 21 ± 9.7 | 12 ± 10.6 | 50 | 100 | 75 | 8 |
| FOR2107-Marburg | 37.1 ± 13.6 | 61.3 | 26.2 ± 12.8 | 19 ± 11.2 | 66 | 78.6 | 63.3 | 244 |
| FOR2107-Münster | 32.4 ± 13 | 59 | 23.6 ± 11.6 | 15.7 ± 12 | 56.4 | 59 | 61.5 | 39 |
| San Francisco | 15.4 ± 1.5 | 57.9 | 12.5 ± 2.7 | 30.2 ± 9.1 | 100 | 78.9 | 0 | 19 |
| Combined | 42.7 ±14.5 | 62.5 | 31 ± 14.2 | 18.5 ± 12.1 | 62.4 | 64.8 | 47.3 | 966 |

**eTable 15:** **Effects of clinical confounds and CM severity on cortical thickness.** For brevity, only the effect of CM severity and clinical variables of each model is shown. For this analysis, remission was defined as BDI≤12. MDD: major depressive disorder, BDI: Beck depression inventory. CM=childhood maltreatment, BDI: Beck depression inventory, FDR=false discovery rate.

| Region | CM severity | | Recurrence | | Antidepressant | | Remission | | BDI | | Age of onset | |
| --- | --- | --- | --- | --- | --- | --- | --- | --- | --- | --- | --- | --- |
|  | Wald χ2 | p_FDR_ | Wald χ2 | p_FDR_ | Wald χ2 | p_FDR_ | Wald χ2 | p_FDR_ | Wald χ2 | p_FDR_ | Wald χ2 | p_FDR_ |
| Overall thickness | 2.702 | 0.100 | 0.062 | 0.803 | 0.014 | 0.907 | 0.018 | 0.894 | 0.302 | 0.583 | 0.460 | 0.498 |
| Banks of superior temporal sulcus | 1.180 | 0.678 | 0.938 | 0.992 | 0.506 | 0.983 | 0.897 | 0.651 | 0.966 | 0.953 | 1.275 | 0.810 |
| Caudal anterior cingulate | 1.079 | 0.678 | 2.471 | 0.992 | 0.059 | 0.983 | 0.100 | 0.807 | 0.112 | 0.953 | 2.736 | 0.810 |
| Caudal middle frontal | 1.812 | 0.668 | 0.017 | 0.992 | 0.141 | 0.983 | 0.791 | 0.651 | 0.047 | 0.953 | 1.129 | 0.810 |
| Cuneus | 0.204 | 0.846 | 0.161 | 0.992 | 2.455 | 0.983 | 0.115 | 0.807 | 0.215 | 0.953 | 0.156 | 0.850 |
| Entorhinal cortex | 0.021 | 0.946 | 1.675 | 0.992 | 2.318 | 0.983 | 2.013 | 0.651 | 0.556 | 0.953 | 1.064 | 0.810 |
| Frontal pole | 0.236 | 0.846 | 0.065 | 0.992 | 0.155 | 0.983 | 0.889 | 0.651 | 0.392 | 0.953 | 0.050 | 0.850 |
| Fusiform | 0.012 | 0.946 | 0.003 | 0.992 | 0.423 | 0.983 | 2.513 | 0.651 | 0.180 | 0.953 | 0.075 | 0.850 |
| Inferior parietal | 1.723 | 0.668 | 0.029 | 0.992 | 0.043 | 0.983 | 1.155 | 0.651 | 0.076 | 0.953 | 0.274 | 0.850 |
| Inferior temporal | 1.584 | 0.668 | 1.687 | 0.992 | 0.893 | 0.983 | 1.969 | 0.651 | 0.093 | 0.953 | 1.239 | 0.810 |
| Insula | 0.008 | 0.946 | 0.077 | 0.992 | 0.000 | 0.983 | 1.326 | 0.651 | 0.999 | 0.953 | 0.261 | 0.850 |
| Isthmus of cingulate | 0.185 | 0.846 | 0.996 | 0.992 | 0.476 | 0.983 | 1.015 | 0.651 | 0.996 | 0.953 | 0.775 | 0.810 |
| Lateral occipital | 0.178 | 0.846 | 0.419 | 0.992 | 0.001 | 0.983 | 0.426 | 0.728 | 0.164 | 0.953 | 0.637 | 0.828 |
| Lateral orbitofrontal | 0.007 | 0.946 | 0.318 | 0.992 | 0.228 | 0.983 | 0.171 | 0.796 | 0.040 | 0.953 | 0.080 | 0.850 |
| Lingual | 0.152 | 0.846 | 0.001 | 0.992 | 0.270 | 0.983 | 1.511 | 0.651 | 0.305 | 0.953 | 0.096 | 0.850 |
| Medial orbitofrontal | 0.406 | 0.846 | 0.434 | 0.992 | 0.376 | 0.983 | 0.856 | 0.651 | 3.075 | 0.953 | 0.886 | 0.810 |
| Middle temporal | 3.190 | 0.668 | 0.000 | 0.992 | 0.050 | 0.983 | 0.762 | 0.651 | 0.087 | 0.953 | 0.404 | 0.850 |
| Paracentral | 1.529 | 0.668 | 0.034 | 0.992 | 0.006 | 0.983 | 1.585 | 0.651 | 0.122 | 0.953 | 0.180 | 0.850 |
| Parahippocampal | 4.293 | 0.668 | 0.212 | 0.992 | 0.490 | 0.983 | 1.096 | 0.651 | 0.663 | 0.953 | 0.845 | 0.810 |
| IFG pars opercularis | 1.775 | 0.668 | 0.620 | 0.992 | 0.262 | 0.983 | 0.196 | 0.796 | 0.122 | 0.953 | 0.766 | 0.810 |
| IFG pars orbitalis | 0.832 | 0.764 | 0.000 | 0.992 | 0.035 | 0.983 | 0.907 | 0.651 | 0.073 | 0.953 | 0.055 | 0.850 |
| IFG pars triangularis | 2.298 | 0.668 | 1.049 | 0.992 | 0.928 | 0.983 | 1.373 | 0.651 | 0.012 | 0.975 | 2.690 | 0.810 |
| Pericalcarine | 0.764 | 0.764 | 0.780 | 0.992 | 1.182 | 0.983 | 0.443 | 0.728 | 0.333 | 0.953 | 0.159 | 0.850 |
| Postcentral | 1.759 | 0.668 | 0.014 | 0.992 | 0.479 | 0.983 | 0.076 | 0.807 | 0.113 | 0.953 | 1.482 | 0.810 |
| Posterior cingulate | 1.172 | 0.678 | 0.691 | 0.992 | 0.035 | 0.983 | 0.285 | 0.771 | 1.440 | 0.953 | 0.000 | 0.999 |
| Precentral | 0.262 | 0.846 | 0.594 | 0.992 | 0.245 | 0.983 | 0.602 | 0.709 | 0.008 | 0.975 | 0.484 | 0.828 |
| Precuneus | 1.229 | 0.678 | 0.263 | 0.992 | 0.016 | 0.983 | 0.833 | 0.651 | 0.005 | 0.975 | 0.049 | 0.850 |
| Rostral anteriorcingulate | 0.604 | 0.825 | 1.088 | 0.992 | 0.615 | 0.983 | 0.962 | 0.651 | 1.005 | 0.953 | 0.979 | 0.810 |
| Rostral middlefrontal | 0.403 | 0.846 | 0.242 | 0.992 | 0.024 | 0.983 | 0.258 | 0.771 | 0.266 | 0.953 | 1.246 | 0.810 |
| Superior frontal | 0.330 | 0.846 | 0.655 | 0.992 | 0.043 | 0.983 | 0.076 | 0.807 | 0.121 | 0.953 | 1.101 | 0.810 |
| Superior parietal | 0.481 | 0.846 | 0.045 | 0.992 | 1.498 | 0.983 | 0.873 | 0.651 | 0.001 | 0.981 | 0.557 | 0.828 |
| Superior temporal | 0.022 | 0.946 | 0.362 | 0.992 | 0.499 | 0.983 | 0.042 | 0.837 | 0.160 | 0.953 | 1.712 | 0.810 |
| Supramarginal | 3.422 | 0.668 | 0.038 | 0.992 | 0.374 | 0.983 | 0.382 | 0.730 | 0.062 | 0.953 | 1.622 | 0.810 |
| Temporal pole | 0.005 | 0.946 | 0.008 | 0.992 | 0.405 | 0.983 | 0.434 | 0.728 | 0.061 | 0.953 | 0.498 | 0.828 |
| Transverse temporal | 2.994 | 0.668 | 0.415 | 0.992 | 0.025 | 0.983 | 1.311 | 0.651 | 0.606 | 0.953 | 0.070 | 0.850 |

**eTable 16:** **Effects of clinical confounds and CM type on cortical thickness.** For brevity, only the effect of CM severity and clinical variables of each model is shown. For this analysis, remission was defined as BDI≤12. MDD: major depressive disorder, BDI: Beck depression inventory. CM=childhood maltreatment, BDI: Beck depression inventory, FDR=false discovery rate.

| Region | CM type | | Recurrence | | Antidepressant | | Remission | | BDI | | Age of onset | |
| --- | --- | --- | --- | --- | --- | --- | --- | --- | --- | --- | --- | --- |
|  | Wald χ2 | p_FDR_ | Wald χ2 | p_FDR_ | Wald χ2 | p_FDR_ | Wald χ2 | p_FDR_ | Wald χ2 | p_FDR_ | Wald χ2 | p_FDR_ |
| Overall thickness | 6.26 | 0.1 | 0.069 | 0.793 | 0.007 | 0.932 | 0 | 0.995 | 0.236 | 0.627 | 0.708 | 0.4 |
| Banks of superior temporal sulcus | 1.2 | 0.818 | 0.966 | 0.98 | 0.071 | 0.983 | 0.891 | 0.737 | 0.974 | 0.963 | 0.957 | 0.782 |
| Caudal anterior cingulate | 1.573 | 0.808 | 2.258 | 0.98 | 0.045 | 0.983 | 0.103 | 0.82 | 0.271 | 0.963 | 2.377 | 0.782 |
| Caudal middle frontal | 5.883 | 0.473 | 0.072 | 0.98 | 0.074 | 0.983 | 0.559 | 0.737 | 0.084 | 0.963 | 1.079 | 0.782 |
| Cuneus | 2.23 | 0.715 | 0.072 | 0.98 | 2.269 | 0.983 | 0.11 | 0.82 | 0.273 | 0.963 | 0.094 | 0.908 |
| Entorhinal cortex | 1.298 | 0.818 | 1.953 | 0.98 | 2.493 | 0.983 | 1.561 | 0.737 | 0.618 | 0.963 | 1.456 | 0.782 |
| Frontal pole | 4.284 | 0.563 | 0.031 | 0.98 | 0.262 | 0.983 | 0.672 | 0.737 | 0.291 | 0.963 | 0.022 | 0.908 |
| Fusiform | 5.321 | 0.473 | 0.004 | 0.98 | 0.496 | 0.983 | 2.23 | 0.737 | 0.332 | 0.963 | 0.027 | 0.908 |
| Inferior parietal | 0.962 | 0.835 | 0.013 | 0.98 | 0.061 | 0.983 | 1.025 | 0.737 | 0.151 | 0.963 | 0.378 | 0.873 |
| Inferior temporal | 3.418 | 0.563 | 1.471 | 0.98 | 0.837 | 0.983 | 1.676 | 0.737 | 0.145 | 0.963 | 1.313 | 0.782 |
| Insula | 3.131 | 0.575 | 0.078 | 0.98 | 0.017 | 0.983 | 1.031 | 0.737 | 0.591 | 0.963 | 0.147 | 0.908 |
| Isthmus of cingulate | 1.13 | 0.818 | 1.018 | 0.98 | 0.613 | 0.983 | 1.025 | 0.737 | 0.975 | 0.963 | 0.902 | 0.782 |
| Lateral occipital | 1.594 | 0.808 | 0.326 | 0.98 | 0 | 0.999 | 0.385 | 0.768 | 0.097 | 0.963 | 0.677 | 0.864 |
| Lateral orbitofrontal | 3.294 | 0.563 | 0.445 | 0.98 | 0.331 | 0.983 | 0.103 | 0.82 | 0.1 | 0.963 | 0.067 | 0.908 |
| Lingual | 3.533 | 0.563 | 0.02 | 0.98 | 0.279 | 0.983 | 1.362 | 0.737 | 0.309 | 0.963 | 0.061 | 0.908 |
| Medial orbitofrontal | 0.322 | 0.956 | 0.425 | 0.98 | 0.305 | 0.983 | 0.905 | 0.737 | 3.021 | 0.963 | 0.893 | 0.782 |
| Middle temporal | 3.685 | 0.563 | 0.008 | 0.98 | 0.072 | 0.983 | 0.728 | 0.737 | 0.036 | 0.963 | 0.438 | 0.864 |
| Paracentral | 5.176 | 0.473 | 0.136 | 0.98 | 0.001 | 0.999 | 1.344 | 0.737 | 0.13 | 0.963 | 0.194 | 0.908 |
| Parahippocampal | 3.525 | 0.563 | 0.146 | 0.98 | 0.461 | 0.983 | 1.036 | 0.737 | 0.867 | 0.963 | 0.908 | 0.782 |
| IFG pars opercularis | 7.499 | 0.359 | 0.483 | 0.98 | 0.414 | 0.983 | 0.083 | 0.821 | 0.031 | 0.963 | 0.584 | 0.864 |
| IFG pars orbitalis | 5.064 | 0.473 | 0.012 | 0.98 | 0.004 | 0.999 | 0.621 | 0.737 | 0.038 | 0.963 | 0.044 | 0.908 |
| IFG pars triangularis | 3.686 | 0.563 | 0.798 | 0.98 | 0.621 | 0.983 | 1.05 | 0.737 | 0.006 | 0.963 | 2.971 | 0.782 |
| Pericalcarine | 6.942 | 0.359 | 1.238 | 0.98 | 1.075 | 0.983 | 0.423 | 0.768 | 0.254 | 0.963 | 0.11 | 0.908 |
| Postcentral | 7.52 | 0.359 | 0 | 0.994 | 0.589 | 0.983 | 0.014 | 0.933 | 0.385 | 0.963 | 1.144 | 0.782 |
| Posterior cingulate | 1.453 | 0.812 | 0.553 | 0.98 | 0.017 | 0.983 | 0.358 | 0.768 | 1.393 | 0.963 | 0.003 | 0.957 |
| Precentral | 8.403 | 0.359 | 0.895 | 0.98 | 0.211 | 0.983 | 0.331 | 0.768 | 0.137 | 0.963 | 0.285 | 0.908 |
| Precuneus | 6.92 | 0.359 | 0.101 | 0.98 | 0.029 | 0.983 | 0.608 | 0.737 | 0.002 | 0.963 | 0.023 | 0.908 |
| Rostral anteriorcingulate | 2.039 | 0.738 | 1.104 | 0.98 | 0.652 | 0.983 | 0.96 | 0.737 | 1.009 | 0.963 | 0.958 | 0.782 |
| Rostral middlefrontal | 2.44 | 0.689 | 0.2 | 0.98 | 0.027 | 0.983 | 0.168 | 0.82 | 0.29 | 0.963 | 1.207 | 0.782 |
| Superior frontal | 2.685 | 0.655 | 0.545 | 0.98 | 0.029 | 0.983 | 0.19 | 0.82 | 0.216 | 0.963 | 1.032 | 0.782 |
| Superior parietal | 3.991 | 0.563 | 0.121 | 0.98 | 1.599 | 0.983 | 0.635 | 0.737 | 0.011 | 0.963 | 0.481 | 0.864 |
| Superior temporal | 4.795 | 0.489 | 0.252 | 0.98 | 0.358 | 0.983 | 0 | 0.992 | 0.013 | 0.963 | 1.397 | 0.782 |
| Supramarginal | 6.96 | 0.359 | 0.007 | 0.98 | 0.526 | 0.983 | 0.191 | 0.82 | 0.134 | 0.963 | 1.551 | 0.782 |
| Temporal pole | 5.156 | 0.473 | 0.004 | 0.98 | 0.416 | 0.983 | 0.719 | 0.737 | 0.076 | 0.963 | 0.478 | 0.864 |
| Transverse temporal | 9.647 | 0.359 | 0.223 | 0.98 | 0.073 | 0.983 | 0.705 | 0.737 | 0.331 | 0.963 | 0.114 | 0.908 |

**eTable 17:** **Effects of clinical confounds and CM severity on cortical surface.** For brevity, only the effect of CM severity and clinical variables of each model is shown. For this analysis, remission was defined as BDI≤12. MDD: major depressive disorder, BDI: Beck depression inventory. CM=childhood maltreatment, BDI: Beck depression inventory, FDR=false discovery rate.

| Region | CM severity | | Recurrency | | Antidepressant | | Remission | | BDI | | Age of onset | |
| --- | --- | --- | --- | --- | --- | --- | --- | --- | --- | --- | --- | --- |
|  | Wald χ2 | p_FDR_ | Wald χ2 | p_FDR_ | Wald χ2 | p_FDR_ | Wald χ2 | p_FDR_ | Wald χ2 | p_FDR_ | Wald χ2 | p_FDR_ |
| Overall surface | 0.342 | 0.559 | 1.541 | 0.214 | 1.321 | 0.250 | 0.433 | 0.511 | 0.149 | 0.700 | 0.020 | 0.886 |
| Banks of superior temporal sulcus | 0.050 | 0.960 | 4.224 | 0.453 | 1.784 | 0.721 | 2.687 | 0.870 | 1.164 | 0.866 | 0.086 | 0.938 |
| Caudal anterior cingulate | 0.003 | 0.960 | 0.063 | 0.992 | 0.131 | 0.901 | 1.111 | 0.874 | 0.061 | 0.866 | 0.738 | 0.938 |
| Caudal middle frontal | 1.121 | 0.960 | 4.732 | 0.453 | 0.634 | 0.901 | 1.480 | 0.874 | 0.030 | 0.866 | 0.425 | 0.938 |
| Cuneus | 0.187 | 0.960 | 1.084 | 0.748 | 1.558 | 0.721 | 0.066 | 0.874 | 2.966 | 0.866 | 1.025 | 0.938 |
| Entorhinal cortex | 0.003 | 0.960 | 0.374 | 0.933 | 0.409 | 0.901 | 0.711 | 0.874 | 2.436 | 0.866 | 0.663 | 0.938 |
| Frontal pole | 6.333 | 0.408 | 0.048 | 0.992 | 1.659 | 0.721 | 2.319 | 0.870 | 0.367 | 0.866 | 0.123 | 0.938 |
| Fusiform | 0.157 | 0.960 | 5.543 | 0.453 | 4.695 | 0.255 | 3.555 | 0.870 | 0.264 | 0.866 | 0.124 | 0.938 |
| Inferior parietal | 0.042 | 0.960 | 1.910 | 0.631 | 0.007 | 0.976 | 0.997 | 0.874 | 0.089 | 0.866 | 0.040 | 0.938 |
| Inferior temporal | 0.657 | 0.960 | 0.645 | 0.844 | 0.068 | 0.901 | 0.022 | 0.922 | 0.115 | 0.866 | 0.208 | 0.938 |
| Insula | 0.505 | 0.960 | 1.322 | 0.748 | 0.132 | 0.901 | 0.739 | 0.874 | 0.029 | 0.866 | 0.066 | 0.938 |
| Isthmus of cingulate | 4.149 | 0.714 | 1.966 | 0.631 | 0.108 | 0.901 | 0.293 | 0.874 | 2.047 | 0.866 | 0.185 | 0.938 |
| Lateral occipital | 0.004 | 0.960 | 0.094 | 0.992 | 0.644 | 0.901 | 1.414 | 0.874 | 0.137 | 0.866 | 0.418 | 0.938 |
| Lateral orbitofrontal | 0.512 | 0.960 | 0.448 | 0.933 | 0.003 | 0.976 | 0.217 | 0.874 | 1.845 | 0.866 | 0.385 | 0.938 |
| Lingual | 1.035 | 0.960 | 1.245 | 0.748 | 0.664 | 0.901 | 0.083 | 0.874 | 1.835 | 0.866 | 1.036 | 0.938 |
| Medial orbitofrontal | 0.495 | 0.960 | 0.003 | 0.992 | 0.149 | 0.901 | 4.380 | 0.870 | 1.511 | 0.866 | 0.043 | 0.938 |
| Middle temporal | 2.025 | 0.878 | 1.292 | 0.748 | 0.001 | 0.976 | 2.527 | 0.870 | 0.885 | 0.866 | 1.268 | 0.938 |
| Paracentral | 0.004 | 0.960 | 0.018 | 0.992 | 0.083 | 0.901 | 0.008 | 0.929 | 0.160 | 0.866 | 0.000 | 0.991 |
| Parahippocampal | 0.261 | 0.960 | 0.358 | 0.933 | 1.209 | 0.841 | 0.256 | 0.874 | 0.172 | 0.866 | 0.120 | 0.938 |
| IFG pars opercularis | 0.005 | 0.960 | 0.005 | 0.992 | 4.805 | 0.255 | 0.017 | 0.922 | 0.407 | 0.866 | 1.231 | 0.938 |
| IFG pars orbitalis | 0.692 | 0.960 | 2.331 | 0.631 | 0.132 | 0.901 | 0.114 | 0.874 | 0.601 | 0.866 | 0.214 | 0.938 |
| IFG pars triangularis | 0.410 | 0.960 | 0.245 | 0.960 | 3.922 | 0.326 | 0.662 | 0.874 | 0.128 | 0.866 | 0.397 | 0.938 |
| Pericalcarine | 1.133 | 0.960 | 1.037 | 0.748 | 0.313 | 0.901 | 0.082 | 0.874 | 0.507 | 0.866 | 3.109 | 0.938 |
| Postcentral | 0.331 | 0.960 | 0.009 | 0.992 | 2.670 | 0.495 | 0.143 | 0.874 | 1.044 | 0.866 | 0.981 | 0.938 |
| Posterior cingulate | 1.100 | 0.960 | 0.055 | 0.992 | 0.133 | 0.901 | 1.116 | 0.874 | 0.049 | 0.866 | 0.471 | 0.938 |
| Precentral | 0.926 | 0.960 | 0.000 | 0.992 | 7.072 | 0.187 | 0.347 | 0.874 | 0.285 | 0.866 | 1.501 | 0.938 |
| Precuneus | 0.013 | 0.960 | 2.713 | 0.631 | 0.001 | 0.976 | 0.730 | 0.874 | 0.152 | 0.866 | 0.186 | 0.938 |
| Rostral anteriorcingulate | 2.209 | 0.878 | 0.001 | 0.992 | 0.233 | 0.901 | 0.196 | 0.874 | 0.049 | 0.866 | 0.107 | 0.938 |
| Rostral middlefrontal | 0.026 | 0.960 | 0.679 | 0.844 | 6.486 | 0.187 | 0.423 | 0.874 | 0.441 | 0.866 | 1.248 | 0.938 |
| Superior frontal | 0.005 | 0.960 | 0.009 | 0.992 | 0.839 | 0.901 | 0.251 | 0.874 | 0.380 | 0.866 | 0.002 | 0.991 |
| Superior parietal | 0.283 | 0.960 | 0.002 | 0.992 | 2.788 | 0.495 | 0.339 | 0.874 | 1.382 | 0.866 | 0.058 | 0.938 |
| Superior temporal | 0.028 | 0.960 | 2.216 | 0.631 | 0.632 | 0.901 | 0.862 | 0.874 | 0.589 | 0.866 | 0.028 | 0.938 |
| Supramarginal | 0.264 | 0.960 | 2.772 | 0.631 | 0.201 | 0.901 | 1.375 | 0.874 | 0.503 | 0.866 | 0.022 | 0.938 |
| Temporal pole | 3.162 | 0.850 | 0.268 | 0.960 | 0.479 | 0.901 | 0.081 | 0.874 | 0.277 | 0.866 | 0.046 | 0.938 |
| Transverse temporal | 2.115 | 0.878 | 0.669 | 0.844 | 0.382 | 0.901 | 0.187 | 0.874 | 2.777 | 0.866 | 0.033 | 0.938 |

**eTable 18:** **Effects of clinical confounds and CM type on cortical surface.** For brevity, only the effect of CM severity and clinical variables of each model is shown. For this analysis, remission was defined as BDI≤12. MDD: major depressive disorder, BDI: Beck depression inventory. CM=childhood maltreatment, BDI: Beck depression inventory, FDR=false discovery rate.

| Region | CM type | | Recurrency | | Antidepressant | | Remission | | BDI | | Age of onset | |
| --- | --- | --- | --- | --- | --- | --- | --- | --- | --- | --- | --- | --- |
|  | Wald χ2 | p_FDR_ | Wald χ2 | p_FDR_ | Wald χ2 | p_FDR_ | Wald χ2 | p_FDR_ | Wald χ2 | p_FDR_ | Wald χ2 | p_FDR_ |
| Overall surface | 1.943 | 0.584 | 1.801 | 0.180 | 1.300 | 0.254 | 0.353 | 0.553 | 0.276 | 0.600 | 0.025 | 0.874 |
| Banks of superior temporal sulcus | 0.612 | 0.950 | 4.447 | 0.397 | 1.782 | 0.738 | 2.718 | 0.842 | 1.576 | 0.899 | 0.027 | 0.995 |
| Caudal anterior cingulate | 1.293 | 0.861 | 0.043 | 1.000 | 0.123 | 0.880 | 1.009 | 0.846 | 0.041 | 0.922 | 0.873 | 0.995 |
| Caudal middle frontal | 0.164 | 0.983 | 4.641 | 0.397 | 0.653 | 0.880 | 1.412 | 0.846 | 0.003 | 0.979 | 0.661 | 0.995 |
| Cuneus | 3.464 | 0.778 | 1.149 | 0.714 | 1.522 | 0.738 | 0.061 | 0.902 | 3.190 | 0.810 | 0.850 | 0.995 |
| Entorhinal cortex | 5.124 | 0.762 | 0.550 | 0.865 | 0.360 | 0.880 | 0.408 | 0.846 | 2.403 | 0.810 | 0.835 | 0.995 |
| Frontal pole | 4.890 | 0.762 | 0.006 | 1.000 | 1.613 | 0.738 | 2.010 | 0.846 | 0.293 | 0.901 | 0.117 | 0.995 |
| Fusiform | 8.742 | 0.487 | 6.548 | 0.374 | 4.911 | 0.247 | 3.183 | 0.842 | 0.355 | 0.901 | 0.330 | 0.995 |
| Inferior parietal | 2.463 | 0.827 | 2.293 | 0.631 | 0.010 | 0.959 | 1.062 | 0.846 | 0.070 | 0.903 | 0.071 | 0.995 |
| Inferior temporal | 1.158 | 0.861 | 0.564 | 0.865 | 0.046 | 0.940 | 0.075 | 0.902 | 0.086 | 0.903 | 0.197 | 0.995 |
| Insula | 3.929 | 0.762 | 1.678 | 0.680 | 0.176 | 0.880 | 0.513 | 0.846 | 0.001 | 0.979 | 0.133 | 0.995 |
| Isthmus of cingulate | 3.981 | 0.762 | 1.640 | 0.680 | 0.142 | 0.880 | 0.325 | 0.846 | 2.473 | 0.810 | 0.128 | 0.995 |
| Lateral occipital | 1.134 | 0.861 | 0.160 | 1.000 | 0.702 | 0.880 | 1.268 | 0.846 | 0.167 | 0.903 | 0.466 | 0.995 |
| Lateral orbitofrontal | 3.713 | 0.769 | 0.332 | 0.957 | 0.010 | 0.959 | 0.280 | 0.846 | 2.165 | 0.810 | 0.561 | 0.995 |
| Lingual | 5.733 | 0.762 | 1.374 | 0.689 | 0.671 | 0.880 | 0.047 | 0.902 | 2.145 | 0.810 | 0.943 | 0.995 |
| Medial orbitofrontal | 2.186 | 0.827 | 0.013 | 1.000 | 0.147 | 0.880 | 3.768 | 0.842 | 0.946 | 0.901 | 0.002 | 0.995 |
| Middle temporal | 2.868 | 0.778 | 1.365 | 0.689 | 0.002 | 0.961 | 2.722 | 0.842 | 0.721 | 0.901 | 1.290 | 0.995 |
| Paracentral | 0.437 | 0.961 | 0.014 | 1.000 | 0.062 | 0.940 | 0.024 | 0.905 | 0.075 | 0.903 | 0.011 | 0.995 |
| Parahippocampal | 8.159 | 0.487 | 0.615 | 0.865 | 1.107 | 0.880 | 0.341 | 0.846 | 0.297 | 0.901 | 0.061 | 0.995 |
| IFG pars opercularis | 1.067 | 0.861 | 0.009 | 1.000 | 4.756 | 0.247 | 0.006 | 0.936 | 0.224 | 0.901 | 1.722 | 0.995 |
| IFG pars orbitalis | 2.242 | 0.827 | 2.081 | 0.633 | 0.128 | 0.880 | 0.139 | 0.874 | 0.593 | 0.901 | 0.303 | 0.995 |
| IFG pars triangularis | 1.384 | 0.861 | 0.156 | 1.000 | 3.757 | 0.360 | 0.617 | 0.846 | 0.066 | 0.903 | 0.533 | 0.995 |
| Pericalcarine | 3.969 | 0.762 | 1.100 | 0.714 | 0.218 | 0.880 | 0.134 | 0.874 | 0.673 | 0.901 | 3.066 | 0.995 |
| Postcentral | 1.135 | 0.861 | 0.032 | 1.000 | 2.434 | 0.578 | 0.282 | 0.846 | 1.394 | 0.899 | 0.862 | 0.995 |
| Posterior cingulate | 1.554 | 0.861 | 0.053 | 1.000 | 0.252 | 0.880 | 0.769 | 0.846 | 0.106 | 0.903 | 0.447 | 0.995 |
| Precentral | 3.092 | 0.778 | 0.000 | 1.000 | 7.585 | 0.204 | 0.570 | 0.846 | 0.382 | 0.901 | 1.543 | 0.995 |
| Precuneus | 5.519 | 0.762 | 3.078 | 0.555 | 0.007 | 0.959 | 0.787 | 0.846 | 0.391 | 0.901 | 0.290 | 0.995 |
| Rostral anteriorcingulate | 1.149 | 0.861 | 0.000 | 1.000 | 0.339 | 0.880 | 0.240 | 0.849 | 0.010 | 0.978 | 0.172 | 0.995 |
| Rostral middlefrontal | 3.214 | 0.778 | 0.836 | 0.818 | 6.132 | 0.221 | 0.316 | 0.846 | 0.915 | 0.901 | 0.851 | 0.995 |
| Superior frontal | 2.437 | 0.827 | 0.050 | 1.000 | 0.817 | 0.880 | 0.128 | 0.874 | 0.232 | 0.901 | 0.000 | 0.995 |
| Superior parietal | 1.118 | 0.861 | 0.000 | 1.000 | 2.821 | 0.527 | 0.287 | 0.846 | 1.502 | 0.899 | 0.021 | 0.995 |
| Superior temporal | 4.681 | 0.762 | 2.737 | 0.555 | 0.664 | 0.880 | 0.761 | 0.846 | 0.722 | 0.901 | 0.025 | 0.995 |
| Supramarginal | 2.896 | 0.778 | 3.017 | 0.555 | 0.191 | 0.880 | 1.434 | 0.846 | 0.646 | 0.901 | 0.000 | 0.995 |
| Temporal pole | 3.998 | 0.762 | 0.288 | 0.957 | 0.593 | 0.880 | 0.036 | 0.902 | 0.320 | 0.901 | 0.032 | 0.995 |
| Transverse temporal | 10.509 | 0.487 | 0.387 | 0.956 | 0.307 | 0.880 | 0.421 | 0.846 | 3.152 | 0.810 | 0.061 | 0.995 |
